# A progressive approach to multi-criteria group decision-making: *N*-bipolar hypersoft topology perspective

**DOI:** 10.1371/journal.pone.0304016

**Published:** 2024-05-21

**Authors:** Sagvan Y. Musa, Baravan A. Asaad

**Affiliations:** 1 Department of Mathematics, College of Education, University of Zakho, Zakho, Iraq; 2 Department of Computer Science, College of Science, Cihan University-Duhok, Duhok, Iraq; 3 Department of Mathematics, College of Science, University of Zakho, Zakho, Iraq; Korea National University of Transportation, KOREA, REPUBLIC OF

## Abstract

This paper investigates *N*-bipolar hypersoft topology (*N*-BHST), a novel extension of both the well-established *N*-hypersoft topology (*N*-HST) and hypersoft topology (HST). Deviating significantly from its precursor, the *N*-bipolar hypersoft (*N*-BHS) set, *N*-BHST introduces a multi-opinion approach to decision-making, augmenting robustness and adaptability. This innovative framework addresses identified limitations in *N*-bipolar soft topology (*N*-BST), especially in managing multi-argument approximate functions. The study analyzes various operators (closure, interior, exterior, and boundary) within the *N*-BHST framework, elucidating their interrelationships. Additionally, an examination is carried out on the enhancement of multi-criteria group decision-making (MCGDM) using *N*-BHST, setting it apart from existing models. A numerical example is presented to illustrate its application in real-world decision scenarios.

## 1 Introduction

In the information age, the challenges associated with decision-making have become increasingly complex due to intricate decision-making environments. Expressing attribute values for alternatives has consequently become more difficult. To address this, Zadeh introduced the concept of fuzzy sets [1], offering a convenient way to represent fuzzy information in decision-making scenarios. Alongside this, probability theory has emerged as a crucial tool for handling intricate information in practical issues, extensively explored by numerous scholars [2, 3]. This heightened interest in fuzzy sets has led to various extensions, intuitionistic fuzzy sets [4], and interval-valued intuitionistic fuzzy sets [5].

The rapid growth in the volume and complexity of gathered information in our contemporary society has led to various forms of ambiguity, particularly in addressing complex issues across diverse fields such as engineering, economics, social science, environmental science, and biology. To effectively describe and extract valuable insights from data enveloped in uncertainty, researchers in computer science, mathematics, and related disciplines have explored several theories, including fuzzy set theory [1], vague set theory [6], probability theory, rough set theory [7–11], and interval mathematics [12]. Additionally, the theory of soft set, introduced by Molodtsov [13] in 1999, has emerged as a novel mathematical tool designed to handle vagueness independently of existing methods’ limitations. Soft set has found wide-ranging applications in various fields, including game theory, probability theory, smoothness of functions, Riemann integration, operations research, and Perron integration. Recently, researchers have shown significant interest in soft set and its various extensions [14–22].

## 2 Related works

In the realm of social judgment frameworks, Alcantud and Laruelle [23] introduced a ternary voting framework within the soft set context. Non-binary assessments are also crucial in ranking and rating scenarios, prompting Fatimah et al. [24] to propose an extended soft set model named *N*-soft (*N*-S) set, emphasizing the importance of ordered grades in real-world problems. Building on this, Alcantud et al. [25] extended *N*-S set to the realm of rough set theory. Akram et al. [26] introduced hesitant *N*-S set, while their subsequent work [27] delved into parameter reduction in *N*-S set. The semantics of *N*-S set were explored by Alcantud [28], and Akram et al. [29] further modified *N*-S set to devise fuzzy *N*-S set. Fatimah and Alcantud [30] contributed to the field with the invention of multi-fuzzy *N*-S set. Shabir and Fatima [31] presented *N*-bipolar soft (*N*-BS) set and its application to decision making. Another extension, intuitionistic fuzzy *N*-S set, was introduced by Akram et al. [32], while the notion of bipolar fuzzy *N*-S set was proposed by Akram et al. [33]. Expanding the scope, Mahmood et al. [34] studied complex fuzzy *N*-S set, and Rehman and Mahmood [35] investigated complex intuitionistic fuzzy *N*-S set. Riaz et al. [36] introduced *N*-soft topology (*N*-ST) as an extension of soft topology, exploring its applications in multi-criteria group decision-making. In a related context, Mustafa [37] proposed *N*-BST as an extension of both *N*-ST and soft topology, applying it to multi-criteria group decision-making. These extensions demonstrate the diverse applications and adaptability of soft set in addressing complex scenarios in recent research efforts.

In 2018, Smarandache [38] introduced the concept of hypersoft (HS) sets, offering an improved framework for handling indistinct and uncertain data within models similar to soft sets. HS sets employ a multi-argument approximation function strategy, enhancing adaptability and reliability compared to traditional soft sets. Building on this, Musa and Asaad [39] introduced bipolar hypersoft (BHS) sets demonstrating their applicability in decision-making problems [40]. Their subsequent work delved into the topological structures associated with BHS sets [41, 42]. The exploration of core principles, properties, and operations of HS sets and their extensions has been the focus of extensive investigations [43–47].

Recent investigations into hybrid HS set models underscore a rising interest among researchers in innovative methodologies. These models incorporate standard HS sets with binary evaluations and fuzzy HS sets utilizing real numbers between 0 and 1. Real-world challenges often entail non-binary and discrete data structures. Addressing this, Musa et al. [48] introduced *N*-HS sets, presenting a more comprehensive framework than conventional HS sets. *N*-HS sets integrate a parametrized object characterization based on ordered grades, providing a versatile representation for intricate real-world scenarios. Furthermore, they [49] introduced the notion of *N*-HST, extending the concept from HST [50].

In addition to Musa’s significant contributions to HS sets, their groundbreaking work on the *N*-BHS set [51] is noteworthy. This innovative hybridization of *N*-HS sets and bipolarity settings enhances HS model capabilities, providing nuanced data representation solutions for non-binary and discrete data structures. Building upon *N*-BHS sets, our article extends the exploration into BHS set theory by introducing *N*-BHST. This extension aims to enhance our understanding of structural properties and relationships within the *N*-BHS framework. We further explore the practical implications of *N*-BHST by examining its application in MCGDM. Integrating topological considerations into *N*-BHS set models enriches the toolkit for researchers and practitioners, offering insights and methodologies for tackling decision-making challenges in real-world scenarios.

### 2.1 Motivation

Inspired by the complexities inherent in decision-making scenarios, this manuscript explores the conceptual realm of the *N*-BHST, marking a substantial departure from its forerunner, the *N*-BHS set. Notably, the *N*-BHST introduces a paradigm shift by embracing a multi-opinion approach to decision-making, in contrast to the single-opinion framework utilized by the *N*-BHS set.

In addition to its unique decision-making approach, the *N*-BHST pioneers a fresh line of inquiry aimed at addressing limitations observed in the *N*-BST regarding the handling of multi-argument approximate functions. The manuscript presents a systematic partitioning of attributes into discrete subattribute values through the application of disjoint sets.

### 2.2 Main contributions

The ensuing key contributions underscore our inventive approaches and methodologies:

- Traced the evolution of soft set models from conventional to advanced concepts like HS sets.
- Introduced *N*-BHST as a resilient multi-opinion approach to decision-making.
- Innovatively partitioned attributes into discrete subattribute values, tackling challenges in multi-argument approximations.
- Proposed algorithms within the *N*-BHST framework for accommodating diverse opinions in decision-making.
- Conducted a comparative analysis, establishing *N*-BHST as a versatile approach for critical evaluation factors.
- Explored fuzzy integration in *N*-BHST and outlined plans to extend the study to some novel models, aiming to derive structures for solving real-world problems with uncertainties.

### 2.3 Paper organization

The organization of this paper is outlined as follow: In Section 1, we provide an introduction to the context and motivation behind our research. In Section 2, we review related works, discussing the state-of-the-art literature, identifying gaps, and highlighting the methodologies proposed by other scholars in addressing similar problems. In Section 3, we review HS sets, *N*-HS sets, and *N*-BHS sets covering associated definitions and results. In Section 4, we delve into the extended *N*-HST, labeled as *N*-BHST, and introduce the corresponding operators. In Section 5, we provide details on MCGDM approaches suitable for *N*-BHST. In Section 6, we conduct a comparative analysis between *N*-BHST and relevant existing models. Finally, the conclusion of our study is presented in Section 7.

## 3 Preliminaries

In this section, we delve into the concepts of HS sets, *N*-HS sets, and *N*-BHS sets, providing an overview and presenting relevant findings associated with each.

### 3.1 Hypersoft sets

**Definition 1**. [38] *Let* ¥ *denote a collection of alternatives*, P(¥) *the power set of* ¥. *Consider a*_1_, *a*_2_, …, *a*_*n*_ *as n distinct attributes, each associated with the sets ξ*_1_, *ξ*_2_, …, *ξ*_*n*_, *where ξ*_*i*_ ∩ *ξ*_*j*_ = ∅ *for i* ≠ *j*, *and i*, *j* ∈ {1, 2, …, *n*}. *An HS set is denoted as* $F_{\xi_{1}\times \xi_{2}\times \ldots \times \xi_{n}}$, *where* $F:\xi_{1}\times \xi_{2}\times \ldots \times \xi_{n}\to P\left(¥\right)$.

Let *ξ*_1_ × *ξ*_2_ × … × *ξ*_*n*_ = *ξ* and ξ,ζ⊆E. It’s crucial to highlight that every element in *ξ*, *ζ*, and E is an *n*-tuple element. The representation of the HS set $F_{\xi }$ is as follows:

$$F_{\xi }=\left\{\left(\epsilon ,F\left(\epsilon \right)\right):\epsilon \in \xi \;\text{and}\;F\left(\epsilon \right)\in P\left(¥\right)\right\}.$$

**Definition 2**. [43] *Consider a set of attributes ξ* = {*ϵ*_1_, *ϵ*_2_, …, *ϵ*_*n*_}. *The NOT set of ξ*, *denoted as* ¬*ξ*, *is defined as* ¬*ξ* = {¬*ϵ*_1_, ¬*ϵ*_2_, …, ¬*ϵ*_*n*_}, *where* ¬*ϵ*_*i*_ = *not ϵ*_*i*_ *for i* = 1, 2, …, *n*.

### 3.2 *N*-hypersoft sets

**Definition 3**. [48] *Let* ¥ *denote a collection of alternatives*, *ξ be a set representing attributes, and* ξ⊆E. *Let R be a set of ordered grades, specifically R* = {0, 1, …, *N* − 1}, *where N* ∈ {2, 3, …}. *An N*-*HS set is a pair* $\left(F_{\xi },N\right)$, *where* F:ξ→P(¥×R) *and satisfies the following condition: for each ϵ* ∈ *ξ*, *there exists a unique* $(\eta ,r_{\epsilon })\in ¥\times R$ *such that* $\left(\eta ,r_{\epsilon }\right)\in F\left(\epsilon \right)$ *or, equivalently*, $F\left(\epsilon \right)\left(\eta \right)=r_{\epsilon }$, *where η* ∈ ¥ *and r*_*ϵ*_ ∈ *R*.

The *N*-HS set $\left(F_{\xi },N\right)$ can be expressed as follows:

$$\left(F_{\xi },N\right)=\left\{(\epsilon ,\{\eta ,F(\epsilon )(\eta )\}):\epsilon \in \xi ,\eta \in ¥,\text{and}\;F(\epsilon )(\eta )\in R\right\}.$$

The *N*-HS set $\left(F_{\xi },N\right)$ can be visualized in tabular form, where *r*_*ij*_ denotes $\left(\eta_{i},r_{ij}\right)\in F\left(\epsilon_{j}\right)$ or $F\left(\epsilon_{j}\right)\left(\eta_{i}\right)=r_{ij}$. This tabular representation is depicted in Table 1.

**Table 1:** Tabular representation of an *N*-HS set $\left(F_{\xi },N\right)$.

$\left(F_{\xi },N\right)$	*ϵ*_1_	*ϵ*_2_	…	*ϵ*_*n*_
*η*_1_	*r*_11_	*r*_12_	…	*r*_1*n*_
*η*_2_	*r*_21_	*r*_22_	…	*r*_2*n*_
…	…	…	…	…
*η*_*m*_	*r*_*m*1_	*r*_*m*2_	…	*r*_*mn*_

**Definition 4**. [48] *Suppose* $\left(F_{\xi },N\right)$ *and* $\left(F_{\zeta }^{1},N\right)$ *are two N*-*HS sets. Then*,

1.
$\left(F_{\xi },N\right)$ *is an N*-*HS subset of* $\left(F_{\zeta }^{1},N\right)$, *denoted by* $\left(F_{\xi },N\right)\tilde{\subseteq }\left(F_{\zeta }^{1},N\right)$, *if ξ* ⊆ *ζ and* $F\left(\epsilon \right)\left(\eta \right)\leq F^{1}\left(\epsilon \right)\left(\eta \right)$, ∀ *ϵ* ∈ *ξ and* *η* ∈ ¥.
2.
$\left(F_{\xi },N\right)$ *and* $\left(F_{\zeta }^{1},N\right)$ *are N-HS equal if* $F=F^{1}$ *and ξ* = *ζ*.
3.
$\left(F_{\xi },N\right)$ *is a null N-HS set, denoted by* Λ_0_, *if* ∀ *ϵ* ∈ *ξ and η* ∈ ¥, F(ϵ)(η)=0.
4.
$\left(F_{\xi },N\right)$ *is a whole N-HS set, denoted by* Λ_*N*−1_, *if* ∀ *ϵ* ∈ *ξ and η* ∈ ¥, F(ϵ)(η)=N-1.
5. *The N-HS complement of* $\left(F_{\xi },N\right)$ *is denoted as* ${\left(F_{\xi },N\right)}^{\prime }=\left(F_{\xi }^{\prime },N\right)$ *where* $F^{\prime }\left(\epsilon \right)\left(\eta \right)=\left(N-1)\backslash F(\epsilon )(\eta \right)$.
6. *The N-HS extended union of* $\left(F_{\xi },N\right)$ *and* $\left(F_{\zeta }^{1},N\right)$ *is denoted and defined as* $\left(F_{\xi },N\right){\tilde{\cup }}_{\varepsilon }\left(F_{\zeta }^{1},N\right)=\left(F_{\left(\xi \;\cup \;\zeta \right)}^{2},N\right)$ , *where* ∀ *ϵ* ∈ *ξ* ∪ *ζ and η* ∈ ¥:
  $$\begin{array}{c} F^{2}\left(\epsilon \right)\left(\eta \right)=\{\begin{array}{cc} F(\epsilon )(\eta ) & if\;\epsilon \in \xi \backslash \zeta \\ F^{1}\left(\epsilon \right)\left(\eta \right) & if\;\epsilon \in \zeta \backslash \xi \\ max\{F\left(\epsilon \right)\left(\eta \right),F^{1}\left(\epsilon \right)\left(\eta \right)\} & if\;\epsilon \in \xi \cap \zeta \end{array} \end{array}$$
7. *The N*-*HS extended intersection of* $\left(F_{\xi },N\right)$ *and* $\left(F_{\zeta }^{1},N\right)$ *is denoted and defined as* $\left(F_{\xi },N\right){\tilde{\cap }}_{\varepsilon }\left(F_{\zeta }^{1},N\right)=\left(F_{\left(\xi \;\cup \;\zeta \right)}^{2},N\right)$, *where* ∀ *ϵ* ∈ *ξ* ∪ *ζ and η* ∈ ¥:
  $$\begin{array}{c} F^{2}\left(\epsilon \right)\left(\eta \right)=\{\begin{array}{cc} F(\epsilon )(\eta ) & if\;\epsilon \in \xi \backslash \zeta \\ F^{1}\left(\epsilon \right)\left(\eta \right) & if\;\epsilon \in \zeta \backslash \xi \\ min\{F\left(\epsilon \right)\left(\eta \right),F^{1}\left(\epsilon \right)\left(\eta \right)\} & if\;\epsilon \in \xi \cap \zeta \end{array} \end{array}$$
8. *The N-HS union of* $\left(F_{\xi },N\right)$ *and* $\left(F_{\zeta }^{1},N\right)$ *is denoted and defined as* $\left(F_{\xi },N\right)\tilde{\cup }\left(F_{\zeta }^{1},N\right)=\left(F_{\left(\xi \;\cap \;\zeta \right)}^{2},N\right)$ , *where* ∀ *ϵ* ∈ *ξ* ∩ *ζ and η* ∈ ¥: $F^{2}\left(\epsilon \right)\left(\eta \right)=max\left\{F\left(\epsilon \right)\left(\eta \right),F^{1}\left(\epsilon \right)\left(\eta \right)\right\}$.
9. *The N*-*HS intersection of* $\left(F_{\xi },N\right)$ *and* $\left(F_{\zeta }^{1},N\right)$ *is denoted and defined as* $\left(F_{\xi },N\right)\tilde{\cap }\left(F_{\zeta }^{1},N\right)=\left(F_{\left(\xi \;\cap \;\zeta \right)}^{2},N\right)$, *where* ∀ *ϵ* ∈ *ξ* ∩ *ζ* *and* *η* ∈ ¥: $F^{2}\left(\epsilon \right)\left(\eta \right)=min\left\{F\left(\epsilon \right)\left(\eta \right),F^{1}\left(\epsilon \right)\left(\eta \right)\right\}$.

**Definition 5**. [49] *Suppose* ${\tilde{T}}_{ℵ}$ *is a collection of N*-*HS sets, then* ${\tilde{T}}_{ℵ}$ *is considered an N*-*HST if*:

1. Λ_0_, Λ_*N*−1_ ∈ ${\tilde{T}}_{ℵ}$.
2.
$\left(F_{\xi }^{1},N\right)$ , $\left(F_{\xi }^{2},N\right)\in {\tilde{T}}_{ℵ}\Rightarrow \left(F_{\xi }^{1},N\right)\tilde{\cap }\left(F_{\xi }^{2},N\right)\in {\tilde{T}}_{ℵ}$.
3.
$\left\{\left(F_{\xi }^{i},N\right)|i\in I\right\}\in {\tilde{T}}_{ℵ}\Rightarrow \tilde{\cup }\left\{\left(F_{\xi }^{i},N\right)|i\in I\right\}\in {\tilde{T}}_{ℵ}$ .

*We define* $\left(¥,{\tilde{T}}_{ℵ},\xi ,N\right)$ *as an N*-*hypersoft topological space* (*N-HSTS). The elements of* ${\tilde{T}}_{ℵ}$ *are called N*-*HS open sets, and their complements in the N*-*HS context are referred to as N*-*HS closed sets*.

### 3.3 *N*-bipolar hypersoft sets

**Definition 6**. [51] *Let* ¥ *denote a collection of alternatives*, *ξ be a set representing attributes, and* ξ⊆E. *Let R be a set of ordered grades, specifically R* = {0, 1, …, *N* − 1}, *where N* ∈ {2, 3, …}. *An N-BHS set is a triple* $\left(F_{\xi },G_{\neg \xi },N\right)$, *where* F:ξ→P(¥×R) *and* G:¬ξ→P(¥×R) *and satisfies the following condition: for each ϵ* ∈ *ξ*, *there exists a unique* (*η*, *r*_*ϵ*_), (*η*, *r*_¬*ϵ*_) ∈¥×R *such that* $\left(\eta ,r_{\epsilon }\right)\in F\left(\epsilon \right)$ (*or* $F\left(\epsilon \right)\left(\eta \right)=r_{\epsilon }$) *and* $\left(\eta ,r_{\neg \epsilon }\right)\in G\left(\neg \epsilon \right)$ (*or* $G\left(\neg \epsilon \right)\left(\eta \right)=r_{\neg \epsilon }$), *with the condition that r*_*ϵ*_ + *r*_¬*ϵ*_ ≤ *N* − 1, *where η* ∈ ¥ *and r*_*ϵ*_, *r*_¬*ϵ*_ ∈ *R*.

The *N*-BHS set $\left(F_{\xi },G_{\neg \xi },N\right)$ can be written in this simplified form:

$\left(F_{\xi },G_{\neg \xi },N\right)=\left\{(\epsilon ,\{\eta ,F(\epsilon )(\eta ),G(\neg \epsilon )(\eta )\}):\epsilon \in \xi ,\eta \in ¥,\text{and}\;F(\epsilon )(\eta ),G(\neg \epsilon )(\eta )\in R\right\}$ .

The *N*-BHS set $\left(F_{\xi },G_{\neg \xi },N\right)$ can be represented in tabular form, where $\left(r_{ij}^{+},r_{ij}^{-}\right)$ denotes $\left(\eta_{i},r_{ij}^{+}\right)\in F\left(\epsilon_{j}\right)$ (or $F\left(\epsilon_{j}\right)\left(\eta_{i}\right)=r_{ij}^{+}$), and $\left(\eta_{i},r_{ij}^{-}\right)\in G\left(\neg \epsilon_{j}\right)$ (or $G\left(\neg \epsilon_{j}\right)\left(\eta_{i}\right)=r_{ij}^{-}$). This representation is illustrated in Table 2.

**Table 2:** Tabular form of *N*-BHS set $\left(F_{\xi },G_{\neg \xi },N\right)$.

$\left(F_{\xi },G_{\neg \xi },N\right)$	*ϵ*_1_	*ϵ*_2_	…	*ϵ*_*n*_
*η*_1_	$\left(r_{11}^{+},r_{11}^{-}\right)$	$\left(r_{12}^{+},r_{12}^{-}\right)$	…	$\left(r_{1n}^{+},r_{1n}^{-}\right)$
*η*_2_	$\left(r_{21}^{+},r_{21}^{-}\right)$	$\left(r_{22}^{+},r_{22}^{-}\right)$	…	$\left(r_{2n}^{+},r_{2n}^{-}\right)$
…	…	…	…	…
*η*_*m*_	$\left(r_{m1}^{+},r_{m1}^{-}\right)$	$\left(r_{m2}^{+},r_{m2}^{-}\right)$	…	$\left(r_{mn}^{+},r_{mn}^{-}\right)$

**Definition 7**. [51] *Suppose* $\left(F_{\xi },G_{\neg \xi },N\right)$ *and* $\left(F_{\zeta }^{1},G_{\neg \zeta }^{1},N\right)$ *are two N*-*BHS sets. Then*,

1.
$\left(F_{\xi },G_{\neg \xi },N\right)$ *is an N*-*BHS subset of* $\left(F_{\zeta }^{1},G_{\neg \zeta }^{1},N\right)$, *denoted by* $\left(F_{\xi },G_{\neg \xi },N\right)\tilde{⊑}\left(F_{\zeta }^{1},G_{\neg \zeta }^{1},N\right)$, *if ξ* ⊆ *ζ* *and* $F\left(\epsilon \right)\left(\eta \right)\leq F^{1}\left(\epsilon \right)\left(\eta \right)$ *and* $G^{1}\left(\neg \epsilon \right)\left(\eta \right)\leq G\left(\neg \epsilon \right)\left(\eta \right)$, ∀ *ϵ* ∈ *ξ and η* ∈ ¥.
2.
$\left(F_{\xi },G_{\neg \xi },N\right)$ *and* $\left(F_{\zeta }^{1},G_{\neg \zeta }^{1},N\right)$ *are N*-*BHS equal if* $F=F^{1}$, $G=G^{1}$, *and ξ* = *ζ*.
3.
$\left(F_{\xi },G_{\neg \xi },N\right)$ *is a null N*-*BHS set, denoted by* $\Lambda_{0}^{N-1}$, *if* ∀ *ϵ* ∈ *ξ and η* ∈ ¥, F(ϵ)(η)=0 *and* G(¬ϵ)(η)=N-1.
4.
$\left(F_{\xi },G_{\neg \xi },N\right)$ *is a whole N*-*BHS set, denoted by* $\Lambda_{N-1}^{0}$, *if* ∀ *ϵ* ∈ *ξ* *and* *η* ∈ ¥, F(ϵ)(η)=N-1 *and* G(¬ϵ)(η)=0.
5. *The N*-*BHS complement of* $\left(F_{\xi },G_{\neg \xi },N\right)$ *is* ${\left(F_{\xi },G_{\neg \xi },N\right)}^{c}=\left(F_{\xi }^{c},G_{\neg \zeta }^{c},N\right)$, *where*, ∀ *ϵ* ∈ *ξ and η* ∈ ¥, $F^{c}\left(\epsilon \right)\left(\eta \right)=G(\neg \epsilon )(\eta )$ *and* $G^{c}\left(\neg \epsilon \right)\left(\eta \right)=F(\epsilon )(\eta )$.
6. *The N*-*BHS extended union of* $\left(F_{\xi },G_{\neg \xi },N\right)$ *and* $\left(F_{\zeta }^{1},G_{\neg \zeta }^{1},N\right)$ *is denoted and defined as* $\left(F_{\xi },G_{\neg \xi },N\right){\tilde{⊔}}_{\varepsilon }\left(F_{\zeta }^{1},G_{\neg \zeta }^{1},N\right)=\left(F_{\left(\xi \;\cup \;\zeta \right)}^{2},G_{\neg (\xi \;\cup \;\zeta )}^{2},N\right)$, *where* ∀ *ϵ* ∈ *ξ* ∪ *ζ and η* ∈ ¥:
  $$\begin{array}{c} F^{2}\left(\epsilon \right)\left(\eta \right)=\{\begin{array}{cc} F(\epsilon )(\eta ) & if\;\epsilon \in \xi \backslash \zeta \\ F^{1}\left(\epsilon \right)\left(\eta \right) & if\;\epsilon \in \zeta \backslash \xi \\ max\{F\left(\epsilon \right)\left(\eta \right),F^{1}\left(\epsilon \right)\left(\eta \right)\} & if\;\epsilon \in \xi \cap \zeta \end{array} \end{array}$$
  $$\begin{array}{c} G^{2}\left(\neg \epsilon \right)\left(\eta \right)=\{\begin{array}{cc} G(\neg \epsilon )(\eta ) & if\;\neg \epsilon \in \neg \xi \backslash \neg \zeta \\ G^{1}\left(\neg \epsilon \right)\left(\eta \right) & if\;\neg \epsilon \in \neg \zeta \backslash \neg \xi \\ min\{G\left(\neg \epsilon \right)\left(\eta \right),G^{1}\left(\neg \epsilon \right)\left(\eta \right)\} & if\;\neg \epsilon \in \neg \xi \cap \neg \zeta \end{array} \end{array}$$
7. *The N*-*BHS extended intersection of* $\left(F_{\xi },G_{\neg \xi },N\right)$ *and* $\left(F_{\zeta }^{1},G_{\neg \zeta }^{1},N\right)$ *is denoted and defined as* $\left(F_{\xi },G_{\neg \xi },N\right){\tilde{⊓}}_{\varepsilon }\left(F_{\zeta }^{1},G_{\neg \zeta }^{1},N\right)=\left(F_{\left(\xi \;\cup \;\zeta \right)}^{2},G_{\neg (\xi \;\cup \;\zeta )}^{2},N\right)$, *where* ∀ *ϵ* ∈ *ξ* ∪ *ζ and η* ∈ ¥:
  $$\begin{array}{c} F^{2}\left(\epsilon \right)\left(\eta \right)=\{\begin{array}{cc} F(\epsilon )(\eta ) & if\;\epsilon \in \xi \backslash \zeta \\ F^{1}\left(\epsilon \right)\left(\eta \right) & if\;\epsilon \in \zeta \backslash \xi \\ min\{F\left(\epsilon \right)\left(\eta \right),F^{1}\left(\epsilon \right)\left(\eta \right)\} & if\;\epsilon \in \xi \cap \zeta \end{array} \end{array}$$
  $$\begin{array}{c} G^{2}\left(\neg \epsilon \right)\left(\eta \right)=\{\begin{array}{cc} G(\neg \epsilon )(\eta ) & if\;\neg \epsilon \in \neg \xi \backslash \neg \zeta \\ G^{1}\left(\neg \epsilon \right)\left(\eta \right) & if\;\neg \epsilon \in \neg \zeta \backslash \neg \xi \\ max\{G\left(\neg \epsilon \right)\left(\eta \right),G^{1}\left(\neg \epsilon \right)\left(\eta \right)\} & if\;\neg \epsilon \in \neg \xi \cap \neg \zeta \end{array} \end{array}$$
8. *The N*-*BHS union of* $\left(F_{\xi },G_{\neg \xi },N\right)$ *and* $\left(F_{\zeta }^{1},G_{\neg \zeta }^{1},N\right)$ *is denoted and defined as* $\left(F_{\xi },G_{\neg \xi },N\right)\tilde{⊔}\left(F_{\zeta }^{1},G_{\neg \zeta }^{1},N\right)=\left(F_{\left(\xi \;\cap \;\zeta \right)}^{2},G_{\neg (\xi \;\cap \;\zeta )}^{2},N\right)$, *where* ∀ *ϵ* ∈ *ξ* ∩ *ζ* *and η* ∈ ¥: $F^{2}\left(\epsilon \right)\left(\eta \right)=max\left\{F\left(\epsilon \right)\left(\eta \right),F^{1}\left(\epsilon \right)\left(\eta \right)\right\}$ *and* $G^{2}\left(\neg \epsilon \right)\left(\eta \right)=min\left\{G\left(\neg \epsilon \right)\left(\eta \right),G^{1}\left(\neg \epsilon \right)\left(\eta \right)\right\}$.
9. *The N*-*BHS intersection of* $\left(F_{\xi },G_{\neg \xi },N\right)$ *and* $\left(F_{\zeta }^{1},G_{\neg \zeta }^{1},N\right)$ *is denoted and defined as* $\left(F_{\xi },G_{\neg \xi },N\right)\tilde{⊓}\left(F_{\zeta }^{1},G_{\neg \zeta }^{1},N\right)=\left(F_{\left(\xi \;\cap \;\zeta \right)}^{2},G_{\neg (\xi \;\cap \;\zeta )}^{2},N\right)$, *where* ∀ *ϵ* ∈ *ξ* ∩ *ζ* *and* *η* ∈ ¥: $F^{2}\left(\epsilon \right)\left(\eta \right)=min\left\{F\left(\epsilon \right)\left(\eta \right),F^{1}\left(\epsilon \right)\left(\eta \right)\right\}$ *and* $G^{2}\left(\neg \epsilon \right)\left(\eta \right)=max\left\{G\left(\neg \epsilon \right)\left(\eta \right),G^{1}\left(\neg \epsilon \right)\left(\eta \right)\right\}$.

**Proposition 1**. [51] *Suppose* $\left(F_{\xi },G_{\neg \xi },N\right)$ *and* $\left(F_{\xi }^{1},G_{\neg \xi }^{1},N\right)$ *are two N*-*BHS sets. Then*,

1.
$\left(F_{\xi },G_{\neg \xi },N\right)\tilde{⊑}\Lambda_{N-1}^{0}$ .
2.
$\Lambda_{0}^{N-1}\tilde{⊑}\left(F_{\xi },G_{\neg \xi },N\right)$ .
3.
$\left(F_{\xi },G_{\neg \xi },N\right)\tilde{⊑}\left(F_{\xi },G_{\neg \xi },N\right)\tilde{⊔}\left(F_{\xi }^{1},G_{\neg \xi }^{1},N\right)$ .
4.
$\left(F_{\xi },G_{\neg \xi },N\right)\tilde{⊓}\left(F_{\xi }^{1},G_{\neg \xi }^{1},N\right)\tilde{⊑}\left(F_{\xi },G_{\neg \xi },N\right)$ .
5.
${\left({\left(F_{\xi },G_{\neg \xi },N\right)}^{c}\right)}^{c}=\left(F_{\xi },G_{\neg \xi },N\right)$

6.
$\Lambda_{0}^{N-1}\tilde{⊑}\left(F_{\xi },G_{\neg \xi },N\right)\tilde{⊓}{\left(F_{\xi },G_{\neg \xi },N\right)}^{c}\tilde{⊑}\left(F_{\xi },G_{\neg \xi },N\right)\tilde{⊔}{\left(F_{\xi },G_{\neg \xi },N\right)}^{c}\tilde{⊑}\Lambda_{N-1}^{0}$ .
7.
$(\left(F_{\xi },G_{\neg \xi },N\right)\tilde{⊔}\left(F_{\xi }^{1},G_{\neg \xi }^{1},N\right){)}^{c}={\left(\left(F_{\xi },G_{\neg \xi },N\right)\right)}^{c}\tilde{⊓}{\left(\left(F_{\xi }^{1},G_{\neg \xi }^{1},N\right)\right)}^{c}$ .
8.
$(\left(F_{\xi },G_{\neg \xi },N\right)\tilde{⊓}\left(F_{\xi }^{1},G_{\neg \xi }^{1},N\right){)}^{c}={\left(\left(F_{\xi },G_{\neg \xi },N\right)\right)}^{c}\tilde{⊔}{\left(\left(F_{\xi }^{1},G_{\neg \xi }^{1},N\right)\right)}^{c}$ .

## 4 *N*-bipolar hypersoft topology and its operators

In this section, we present *N*-BHST as an extension of the *N*-HST and HST. Additionally, we explore various operators, including closure, interior, exterior, and boundary, within the framework of *N*-BHST, examining their interrelationships.

**Definition 8**. *Suppose* ${\tilde{ℑ}}_{ℵ}$ *is a collection of N*-*BHS sets, then* ${\tilde{ℑ}}_{ℵ}$ *is considered an N*-*BHST if*:

1.
$\Lambda_{0}^{N-1}$ , $\Lambda_{N-1}^{0}\in {\tilde{ℑ}}_{ℵ}$.
2.
$\left(F_{\xi }^{1},G_{\neg \xi }^{1},N\right)$ , $\left(F_{\xi }^{2},G_{\neg \xi }^{2},N\right)\in {\tilde{ℑ}}_{ℵ}\Rightarrow \left(F_{\xi }^{1},G_{\neg \xi }^{1},N\right)\tilde{⊓}\left(F_{\xi }^{2},G_{\neg \xi }^{2},N\right)\in {\tilde{ℑ}}_{ℵ}$.
3.
$\left\{\left(F_{\xi }^{i},G_{\neg \xi }^{i},N\right)|i\in I\right\}\in {\tilde{ℑ}}_{ℵ}\Rightarrow \tilde{⊔}\left\{\left(F_{\xi }^{i},G_{\neg \xi }^{i},N\right)|i\in I\right\}\in {\tilde{ℑ}}_{ℵ}$ .

*We define* $\left(¥,{\tilde{ℑ}}_{ℵ},\xi ,N\right)$ *as an N*-*bipolar hypersoft topological space* (*N-BHSTS*). *The elements of* ${\tilde{ℑ}}_{ℵ}$ *are called N*-*BHS open sets, and their complements in the N*-*BHS context are referred to as N*-*BHS closed sets*.

**Proposition 2**. *Consider the N-BHSTS* $\left(¥,{\tilde{ℑ}}_{ℵ},\xi ,N\right)$. *Then, the following statements hold:*

1. *The sets* $\Lambda_{0}^{N-1}$ *and* $\Lambda_{N-1}^{0}$ *are N*-*BHS closed*.
2. *The union of any two N*-*BHS closed sets remains a closed set in the N*-*BHS context*.
3. *The intersection of any number of N*-*BHS closed sets remains a closed set in the N*-*BHS context*.

*Proof*. Straightforward.

**Proposition 3**. *Let* $\left(¥,{\tilde{ℑ}}_{ℵ},\xi ,N\right)$ *be an N-BHSTS. Then, the following collections define N-HSTs. In particular*, $\left(¥,{\tilde{T}}_{ℵ},\xi ,N\right)$ *and* $\left(¥,\neg {\tilde{T}}_{ℵ},\neg \xi ,N\right)$ *are N*-*HSTSs*.

*i*. ${\tilde{T}}_{ℵ}=\left\{\left(F_{\xi },N\right):\left(F_{\xi },G_{\neg \xi },N\right)\in {\tilde{ℑ}}_{ℵ}\right\}$.
*ii.* $\neg {\tilde{T}}_{ℵ}=\left\{\left(G_{\neg \xi },N\right):\left(F_{\xi },G_{\neg \xi },N\right)\in {\tilde{ℑ}}_{ℵ}\right\}$ *(provided that* ¥ *and ξ are finite sets)*.

*Proof*. i. Suppose that $\left(¥,{\tilde{ℑ}}_{ℵ},\xi ,N\right)$ is an *N*-BHSTS. Then,

1.
$\Lambda_{0}^{N-1}$ , $\Lambda_{N-1}^{0}\in {\tilde{ℑ}}_{ℵ}$ implies that Λ_0_, Λ_*N*−1_ ∈ ${\tilde{T}}_{ℵ}$.
2. Let $\left(F_{\xi }^{1},N\right)$, $\left(F_{\xi }^{2},N\right)\in {\tilde{T}}_{ℵ}$. Since $\left(F_{\xi }^{1},G_{\neg \xi }^{1},N\right)$, $\left(F_{\xi }^{2},G_{\neg \xi }^{2},N\right)\in {\tilde{ℑ}}_{ℵ}$, then $\left(F_{\xi }^{1},G_{\neg \xi }^{1},N\right)\tilde{⊓}\left(F_{\xi }^{2},G_{\neg \xi }^{2},N\right)\in {\tilde{ℑ}}_{ℵ}$. This implies that $\left(F_{\xi }^{1},N\right)\tilde{\cap }\left(F_{\xi }^{2},N\right)\in {\tilde{T}}_{ℵ}$.
3. Let $\left\{\left(F_{\xi }^{i},N\right):i\in I\right\}\in {\tilde{T}}_{ℵ}$. Since $\left(F_{\xi }^{i},G_{\neg \xi }^{i},N\right)\in {\tilde{ℑ}}_{ℵ}$ for all *i* ∈ *I*, so that ${\tilde{⊔}}_{i\in I}\left(F_{\xi }^{i},G_{\neg \xi }^{i},N\right)\in {\tilde{ℑ}}_{ℵ}$, thus ${\tilde{\cup }}_{i\in I}\left(F_{\xi }^{i},N\right)\in {\tilde{T}}_{ℵ}$.

Hence, ${\tilde{T}}_{ℵ}$ defines an *N*-HST, and $\left(¥,{\tilde{T}}_{ℵ},\xi ,N\right)$ is an *N*-HSTS.

ii. The proof is similar to part (i).

**Corollary 1** *Let* $\left(¥,{\tilde{ℑ}}_{ℵ},\xi ,N\right)$ *be an N-BHSTS. Then, the following collections define N-HST*.

*i*. $\tilde{Ϝ}=\{\left(F_{\xi },N\right):{\left(F_{\xi },G_{\neg \xi },N\right)}^{c}\in {\tilde{ℑ}}_{ℵ}\}$.
*ii*. $\neg \tilde{Ϝ}=\{\left(G_{\neg \xi },N\right):{\left(F_{\xi },G_{\neg \xi },N\right)}^{c}\in {\tilde{ℑ}}_{ℵ}\}$ *(provided that* ¥ *and* E *are finite sets)*.

The following proposition demonstrates when the converse of proposition 3 holds true.

**Proposition 4**. *Let* $\left(¥,{\tilde{T}}_{ℵ},\xi ,N\right)$ *be an N*-*HSTS. Then, the collection* ${\tilde{ℑ}}_{ℵ}$ *consisting of N*-*BHS sets* $\left(F_{\xi },G_{\neg \xi },E\right)$ *such that* $\left(F_{\xi },N\right)\in {\tilde{T}}_{ℵ}$ *and* G(¬ϵ)=(N-1)\F(ϵ) *for all* ¬*ϵ* ∈ ¬*ξ and η* ∈ ¥, *defines an N*-*BHST. In particular*, $\left(¥,{\tilde{ℑ}}_{ℵ},\xi ,N\right)$ *is an N-BHSTS*.

*Proof*. Straightforward.

**Corollary 2**. *Let* $\left(¥,{\tilde{T}}_{ℵ},\xi ,N\right)$ *be an N*-*HSTS. Then, the collection* $\tilde{\tilde{Ϝ}}$ *consisting of N-BHS sets* $\left(F_{\xi },G_{\neg \xi },N\right)$ *such that* ${\left(F_{\xi },N\right)}^{c}\in {\tilde{T}}_{ℵ}$ *and* G(¬ϵ)=(N-1)\F(ϵ) *for all* ¬*ϵ* ∈ ¬*ξ and η* ∈ ¥, *defines an N-BHST*.

**Remark 1**. *If the condition described in proposition 4 is not met, then the following example demonstrates that the converse of proposition 3 is generally not true. This is the case even if the collections* ${\tilde{T}}_{ℵ}$ *and* $\neg {\tilde{T}}_{ℵ}$ *define an N*-*HST*.

**Example 1**. *Let* $¥=\{\eta_{1},\eta_{2}\}$. *Let ξ*_1_ = {*e*_1_, *e*_2_}, *ξ*_2_ = {*e*_3_}, *and ξ*_3_ = {*e*_4_}, *then ξ* = *ξ*_1_ × *ξ*_2_ × *ξ*_3_ = {*ϵ*_1_ = (*e*_1_, *e*_3_, *e*_4_), *ϵ*_2_ = (*e*_2_, *e*_3_, *e*_4_)}. *Let* ${\tilde{T}}_{6}$ = {Λ_0_, Λ_5_, $\left(F_{\xi }^{1},6\right)$, $\left(F_{\xi }^{2},6)\right\}$ *and* $\neg {\tilde{T}}_{6}$ = {Λ_0_, Λ_5_,$\left(G_{\neg \xi }^{1},6\right)$, $\left(G_{\neg \xi }^{2},6)\right\}$ *be two 6-HSTs, where*

$\left(F_{\xi }^{1},6\right)$ = {(*ϵ*_1_, {*η*_1_, 3}, {*η*_2_, 0}), (*ϵ*_2_, {*η*_1_, 2}, {*η*_2_, 4})}.

$\left(F_{\xi }^{2},6\right)$ = {(*ϵ*_1_, {*η*_1_, 3}, {*η*_2_, 5}), (*ϵ*_2_, {*η*_1_, 2}, {*η*_2_, 4})}.

*And*,

$\left(G_{\neg \xi }^{1},6\right)$ = {(*ϵ*_1_, {*η*_1_, 1}, {*η*_2_, 0}), (*ϵ*_2_, {*η*_1_, 1}, {*η*_2_, 0})}.

$\left(G_{\neg \xi }^{2},6\right)$ = {(*ϵ*_1_, {*η*_1_, 2}, {*η*_2_, 0}), (*ϵ*_2_, {*η*_1_, 2}, {*η*_2_, 1})}.

*Then*,

${\tilde{ℑ}}_{6}=\{\Lambda_{0}^{5},\Lambda_{5}^{0},\left(F_{\xi }^{1},G_{\neg \xi }^{1},6\right),\left(F_{\xi }^{2},G_{\neg \xi }^{2},6)\right\}$

*where*

$\left(F_{\xi }^{1},G_{\neg \xi }^{1},6\right)$ = {(*ϵ*_1_, {*η*_1_, 3, 1}, {*η*_2_, 0, 0}), (*ϵ*_2_, {*η*_1_, 2, 1}, {*η*_2_, 4, 0})}.

$\left(F_{\xi }^{2},G_{\neg \xi }^{2},6\right)$ = {(*ϵ*_1_, {*η*_1_, 3, 2}, {*η*_2_, 5, 0}), (*ϵ*_2_, {*η*_1_, 2, 2}, {*η*_2_, 4, 1})}.

*If we take*

$\left(F_{\xi }^{1},G_{\neg \xi }^{1},6\right)\tilde{⊓}\left(F_{\xi }^{2},G_{\neg \xi }^{2},6\right)=\left(F_{\xi },G_{\neg \xi },6\right)$ ,

*then*,

$\left(F_{\xi },G_{\neg \xi },6\right)$ = {(*ϵ*_1_, {*η*_1_, 3, 2}, {*η*_2_, 0, 0}), (*ϵ*_2_, {*η*_1_, 2, 2}, {*η*_2_, 4, 1})}.

*But*, $\left(F_{\xi },G_{\neg \xi },6\right)\notin {\tilde{ℑ}}_{6}$. *Hence*, ${\tilde{ℑ}}_{6}$ *is not a 6-BHST*.

**Definition 9**. *Let* ¥ *denote a set of alternatives, ξ be a set representing attributes, and R* = {0, 1, …, *N* − 1} *be a set representing ordered grades, where N* ∈ {2, 3, …}. *The collection* ${\tilde{ℑ}}_{ℵ}=\left\{\Lambda_{0}^{N-1},\Lambda_{N-1}^{0}\right\}$ *is referred to as an N*-*BHS indiscrete topology*.

**Definition 10**. *Let* ¥ *represent a set of alternatives, ξ be a set representing attributes, R* = {0, 1, …, *N* − 1} *be a set representing ordered grades, where N* ∈ {2, 3, …}, *and* ${\tilde{ℑ}}_{ℵ}$ *be a collection of all N*-*BHS sets that can be defined over* ¥. *Then*, ${\tilde{ℑ}}_{ℵ}$ *is termed an N*-*BHS discrete topology*.

**Proposition 5**. *Let* $\left(¥,{\tilde{ℑ}}_{{ℵ}_{1}},\xi ,N\right)$ *and* $\left(¥,{\tilde{ℑ}}_{{ℵ}_{2}},\xi ,N\right)$ *be two N*-*BHSTSs, then* $(¥,{\tilde{ℑ}}_{{ℵ}_{1}}\cap {\tilde{ℑ}}_{{ℵ}_{2}},$ *ξ*, *N*) *is an N*-*BHSTS*.

*Proof.*

1.
$\Lambda_{0}^{N-1},\Lambda_{N-1}^{0}\in {\tilde{ℑ}}_{{ℵ}_{1}}\cap {\tilde{ℑ}}_{{ℵ}_{2}}$ .
2. Let $\left(F_{\xi }^{1},G_{\neg \xi }^{1},N\right),\left(F_{\xi }^{2},G_{\neg \xi }^{2},N\right)\in {\tilde{ℑ}}_{{ℵ}_{1}}\cap {\tilde{ℑ}}_{{ℵ}_{2}}$. Then, $\left(F_{\xi }^{1},G_{\neg \xi }^{1},N\right),\left(F_{\xi }^{2},G_{\neg \xi }^{2},N\right)\in {\tilde{ℑ}}_{{ℵ}_{1}}$ and $\left(F_{\xi }^{1},G_{\neg \xi }^{1},N\right),\left(F_{\xi }^{2},G_{\neg \xi }^{2},N\right)\in {\tilde{ℑ}}_{{ℵ}_{2}}$. Since $\left(F_{\xi }^{1},G_{\neg \xi }^{1},N\right)\tilde{\cap }\left(F_{\xi }^{2},G_{\neg \xi }^{2},N\right)\in {\tilde{ℑ}}_{{ℵ}_{1}}$ and $\left(F_{\xi }^{1},G_{\neg \xi }^{1},N\right)\tilde{\cap }\left(F_{\xi }^{2},G_{\neg \xi }^{2},N\right)\in {\tilde{ℑ}}_{{ℵ}_{2}}$. Then, $\left(F_{\xi }^{1},G_{\neg \xi }^{1},N\right)\tilde{\cap }\left(F_{\xi }^{2},G_{\neg \xi }^{2},N\right)\in {\tilde{ℑ}}_{{ℵ}_{1}}\cap {\tilde{ℑ}}_{{ℵ}_{2}}$.
3. Let $\left\{\left(F_{\xi }^{i},G_{\neg \xi }^{i},N\right)|i\in I)\right\}\in {\tilde{ℑ}}_{{ℵ}_{1}}\cap {\tilde{ℑ}}_{{ℵ}_{2}}$. Then, $\left(F_{\xi }^{i},G_{\neg \xi }^{i},N\right)\in {\tilde{ℑ}}_{{ℵ}_{1}}$ and $\left(F_{\xi }^{i},G_{\neg \xi }^{i},N\right)\in {\tilde{ℑ}}_{{ℵ}_{2}}$, for all *i* ∈ *I*, and so ${\tilde{⊔}}_{i\in I}\left(F_{\xi }^{i},G_{\neg \xi }^{i},N\right)\in {\tilde{ℑ}}_{{ℵ}_{1}}$ and ${\tilde{⊔}}_{i\in I}\left(F_{\xi }^{i},G_{\neg \xi }^{i},N\right)\in {\tilde{ℑ}}_{{ℵ}_{2}}$. Therefore, ${\tilde{⊔}}_{i\in I}\left(F_{\xi }^{i},G_{\neg \xi }^{i},N\right)\in {\tilde{ℑ}}_{{ℵ}_{1}}\cap {\tilde{ℑ}}_{{ℵ}_{2}}$.

Thus, ${\tilde{ℑ}}_{{ℵ}_{1}}\cap {\tilde{ℑ}}_{{ℵ}_{2}}$ defines an *N*-BHST and $(¥,{\tilde{ℑ}}_{{ℵ}_{1}}\cap {\tilde{ℑ}}_{{ℵ}_{2}},$ *ξ*, *N*) is an *N*-BHSTS.

**Remark 2**. *The union of two N*-*BHSTs may not be an N*-*BHST*.

**Example 2**. *Let* $¥=\{\eta_{1},\eta_{2}\}$. *Let ξ*_1_ = {*e*_1_, *e*_2_}, *ξ*_2_ = {*e*_3_}, *and ξ*_3_ = {*e*_4_}, *then ξ* = *ξ*_1_ × *ξ*_2_ × *ξ*_3_ = {*ϵ*_1_ = (*e*_1_, *e*_3_, *e*_4_), *ϵ*_2_ = (*e*_2_, *e*_3_, *e*_4_)}. *Let* ${\tilde{ℑ}}_{6}=\{\Lambda_{0}^{5},\Lambda_{5}^{0},\left(F_{\xi }^{1},G_{\neg \xi }^{1},6\right),\left(F_{\xi }^{2},G_{\neg \xi }^{2},6\right),\left(F_{\xi }^{3},G_{\neg \xi }^{3},6\right),\left(F_{\xi }^{4},G_{\neg \xi }^{4},6)\right\}$ *and* ${\tilde{T}}_{6}=\{\Lambda_{0}^{5},\Lambda_{5}^{0},\left(H_{\xi }^{1},K_{\neg \xi }^{1},6\right),\left(H_{\xi }^{2},K_{\neg \xi }^{2},6\right),\left(H_{\xi }^{3},K_{\neg \xi }^{3},6\right),\left(H_{\xi }^{4},K_{\neg \xi }^{4},6)\right\}$ *be two 6-BHSTs where*

$\left(F_{\xi }^{1},G_{\neg \xi }^{1},6\right)$ = {(*ϵ*_1_, {*η*_1_, 5, 1}, {*η*_2_, 4, 1}), (*ϵ*_2_, {*η*_1_, 0, 3}, {*η*_2_, 2, 1})}.

$\left(F_{\xi }^{2},G_{\neg \xi }^{2},6\right)$ = {(*ϵ*_1_, {*η*_1_, 4, 2}, {*η*_2_, 4, 0}), (*ϵ*_2_, {*η*_1_, 0, 0}, {*η*_2_, 0, 5})}.

$\left(F_{\xi }^{3},G_{\neg \xi }^{3},6\right)$ = {(*ϵ*_1_, {*η*_1_, 4, 2}, {*η*_2_, 4, 1}), (*ϵ*_2_, {*η*_1_, 0, 3}, {*η*_2_, 0, 5})}.

$\left(F_{\xi }^{4},G_{\neg \xi }^{4},6\right)$ = {(*ϵ*_1_, {*η*_1_, 5, 1}, {*η*_2_, 4, 0}), (*ϵ*_2_, {*η*_1_, 0, 0}, {*η*_2_, 2, 1})}.

*And*

$\left(H_{\xi }^{1},K_{\neg \xi }^{1},6\right)$ = {(*ϵ*_1_, {*η*_1_, 2, 2}, {*η*_2_, 1, 4}), (*ϵ*_2_, {*η*_1_, 3, 1}, {*η*_2_, 4, 1})}.

$\left(H_{\xi }^{2},K_{\neg \xi }^{2},6\right)$ = {(*ϵ*_1_, {*η*_1_, 1, 1}, {*η*_2_, 0, 5}), (*ϵ*_2_, {*η*_1_, 0, 4}, {*η*_2_, 0, 3})}.

$\left(H_{\xi }^{3},K_{\neg \xi }^{3},6\right)$ = {(*ϵ*_1_, {*η*_1_, 1, 2}, {*η*_2_, 0, 5}), (*ϵ*_2_, {*η*_1_, 0, 4}, {*η*_2_, 0, 3})}.

$\left(H_{\xi }^{4},K_{\neg \xi }^{4},6\right)$ = {(*ϵ*_1_, {*η*_1_, 2, 1}, {*η*_2_, 1, 4}), (*ϵ*_2_, {*η*_1_, 3, 1}, {*η*_2_, 4, 1})}.

*Then*, ${\tilde{ℑ}}_{6}⊔{\tilde{T}}_{6}=\{\Lambda_{0}^{5},\Lambda_{5}^{0}$, $\left(F_{\xi }^{1},G_{\neg \xi }^{1},6\right),\left(F_{\xi }^{2},G_{\neg \xi }^{2},6\right),\left(F_{\xi }^{3},G_{\neg \xi }^{3},6\right),\left(F_{\xi }^{4},G_{\neg \xi }^{4},6\right),\left(H_{\xi }^{1},K_{\neg \xi }^{1},6\right),\left(H_{\xi }^{2},K_{\neg \xi }^{2},6\right),\left(H_{\xi }^{3},K_{\neg \xi }^{3},6\right),\left(H_{\xi }^{4},K_{\neg \xi }^{4},6)\right\}$.

*If we take*

$\left(F_{\xi }^{1},G_{\neg \xi }^{1},6\right)\tilde{⊔}\left(H_{\xi }^{1},K_{\neg \xi }^{1},6\right)=\left(O_{\xi },S_{\neg \xi },6\right)$ .

*Then*,

$\left(O_{\xi },S_{\neg \xi },6\right)$ = {(*ϵ*_1_, {*η*_1_, 5, 1}, {*η*_2_, 4, 1}), (*ϵ*_2_, {*η*_1_, 3, 1}, {*η*_2_, 4, 1})}.

*But*, $\left(O_{\xi },S_{\neg \xi },6\right)\notin {\tilde{ℑ}}_{6}\cup {\tilde{T}}_{6}$. *Hence*, ${\tilde{ℑ}}_{6}\cup {\tilde{T}}_{6}$ *is not a 6-BHST. Therefore, in general, the union of two N*-*BHSTs may not be an N*-*BHST*.

**Definition 11**. *Let* $\left(¥,{\tilde{ℑ}}_{ℵ},\xi ,N\right)$ *be an N*-*BHSTS and* $\left(F_{\xi },G_{\neg \xi },N\right)$ *be an N*-*BHS set. The intersection of all N*-*BHS closed supersets of* $\left(F_{\xi },G_{\neg \xi },N\right)$ *is called an N*-*BHS closure of* $\left(F_{\xi },G_{\neg \xi },N\right)$ *and is denoted by* $\overset{˘}{c}\left(\left(F_{\xi },G_{\neg \xi },N\right)\right)$.

*In other words*, $\overset{˘}{c}\left(\left(F_{\xi },G_{\neg \xi },N\right)\right)=\tilde{⊓}\{\left(H_{\xi },K_{\neg \xi },N\right)|{\left(H_{\xi },K_{\neg \xi },N\right)}^{c}\in {\tilde{ℑ}}_{ℵ},\left(H_{\xi },K_{\neg \xi },N\right)\tilde{⊑}\left(F_{\xi },G_{\neg \xi },N)\right\}$.

**Remark 3**. *Let* $\left(¥,{\tilde{ℑ}}_{ℵ},\xi ,N\right)$ *be an N*-*BHSTS and* $\left(F_{\xi },G_{\neg \xi },N\right)$ *be an N*-*BHS set. Then*,

1.
$\overset{˘}{c}\left(\left(F_{\xi },G_{\neg \xi },N\right)\right)$ *is the smallest N-BHS closed set containing* $\left(F_{\xi },G_{\neg \xi },N\right)$ .
2.
$\left(F_{\xi },G_{\neg \xi },N\right)$ *is an N-BHS closed set if and only if* $\left(F_{\xi },G_{\neg \xi },N\right)=\overset{˘}{c}\left(\left(F_{\xi },G_{\neg \xi },N\right)\right)$ .

**Example 3**. *Let* $¥=\{\eta_{1},\eta_{2}\}$. *Let ξ*_1_ = {*e*_1_, *e*_2_}, *ξ*_2_ = {*e*_3_}, *and* *ξ*_3_ = {*e*_4_}, *then ξ* = *ξ*_1_ × *ξ*_2_ × *ξ*_3_ = {*ϵ*_1_ = (*e*_1_, *e*_3_, *e*_4_), *ϵ*_2_ = (*e*_2_, *e*_3_, *e*_4_)}. *Let* ${\tilde{ℑ}}_{6}=\{\Lambda_{0}^{5},\Lambda_{5}^{0},\left(F_{\xi }^{1},G_{\neg \xi }^{1},6\right),\left(F_{\xi }^{2},G_{\neg \xi }^{2},6\right),\left(F_{\xi }^{3},G_{\neg \xi }^{3},6\right),\left(F_{\xi }^{4},G_{\neg \xi }^{4},6)\right\}$ *be a 6-BHST, where*

$\left(F_{\xi }^{1},G_{\neg \xi }^{1},6\right)$ = {(*ϵ*_1_, {*η*_1_, 4, 1}, {*η*_2_, 2, 3}), (*ϵ*_2_, {*η*_1_, 1, 2}, {*η*_2_, 5, 0})}.

$\left(F_{\xi }^{2},G_{\neg \xi }^{2},6\right)$ = {(*ϵ*_1_, {*η*_1_, 2, 2}, {*η*_2_, 3, 1}), (*ϵ*_2_, {*η*_1_, 1, 4}, {*η*_2_, 0, 3})}.

$\left(F_{\xi }^{3},G_{\neg \xi }^{3},6\right)$ = {(*ϵ*_1_, {*η*_1_, 2, 2}, {*η*_2_, 2, 3}), (*ϵ*_2_, {*η*_1_, 1, 4}, {*η*_2_, 0, 3})}.

$\left(F_{\xi }^{4},G_{\neg \xi }^{4},6\right)$ = {(*ϵ*_1_, {*η*_1_, 4, 1}, {*η*_2_, 3, 1}), (*ϵ*_2_, {*η*_1_, 1, 2}, {*η*_2_, 5, 0})}.

*Let* $\left(F_{\xi },G_{\neg \xi },6\right)$ *be any 6-BHS set defined as follows*:

$\left(F_{\xi },G_{\neg \xi },6\right)$ = {(*ϵ*_1_, {*η*_1_, 0, 5}, {*η*_2_, 2, 2}), (*ϵ*_2_, {*η*_1_, 1, 4}, {*η*_2_, 0, 5})}.

*Then*,

$\overset{˘}{c}\left(\left(F_{\xi },G_{\neg \xi },6\right)\right)$ = {(*ϵ*_1_, {*η*_1_, 1, 4}, {*η*_2_, 3, 2}), (*ϵ*_2_, {*η*_1_, 2, 1}, {*η*_2_, 0, 5})}.

**Proposition 6**. *Let* $\left(¥,{\tilde{ℑ}}_{ℵ},\xi ,N\right)$ *be an N*-*BHSTS and let* $\left(F_{\xi }^{1},G_{\neg \xi }^{1},N\right)$ *and* $\left(F_{\xi }^{2},G_{\neg \xi }^{2},N\right)$ *be two N-BHS sets. Then*,

1.
$\overset{˘}{c}\left(\Lambda_{0}^{N-1}\right)=\Lambda_{0}^{N-1}$ *and* $\overset{˘}{c}\left(\Lambda_{N-1}^{0}\right)=\Lambda_{N-1}^{0}$.
2.
$\left(F_{\xi }^{1},G_{\neg \xi }^{1},N\right)\tilde{⊑}\overset{˘}{c}\left(\left(F_{\xi }^{1},G_{\neg \xi }^{1},N\right)\right)$ .
3.
$\left(F_{\xi }^{1},G_{\neg \xi }^{1},N\right)\tilde{⊑}\left(F_{\xi }^{2},G_{\neg \xi }^{2},N\right)$ *implies* $\overset{˘}{c}\left(\left(F_{\xi }^{1},G_{\neg \xi }^{1},N\right)\right)\tilde{⊑}\overset{˘}{c}\left(\left(F_{\xi }^{2},G_{\neg \xi }^{2},N\right)\right)$.
4.
$\overset{˘}{c}\left(\left(F_{\xi }^{1},G_{\neg \xi }^{1},N\right)\;\tilde{⊔}\;\left(F_{\xi }^{2},G_{\neg \xi }^{2},N\right)\right)=\overset{˘}{c}\left(\left(F_{\xi }^{1},G_{\neg \xi }^{1},N\right)\right)\tilde{⊔}\overset{˘}{c}\left(\left(F_{\xi }^{2},G_{\neg \xi }^{2},N\right)\right)$ .
5.
$\overset{˘}{c}\left(\left(F_{\xi }^{1},G_{\neg \xi }^{1},N\right)\;\tilde{⊓}\;\left(F_{\xi }^{2},G_{\neg \xi }^{2},N\right)\right)\tilde{⊑}\overset{˘}{c}\left(\left(F_{\xi }^{1},G_{\neg \xi }^{1},N\right)\right)\tilde{⊓}\overset{˘}{c}\left(\left(F_{\xi }^{2},G_{\neg \xi }^{2},N\right)\right)$ .
6.
$\overset{˘}{c}\left(\overset{˘}{c}\left(\left(F_{\xi }^{1},G_{\neg \xi }^{1},N\right)\right)\right)=\overset{˘}{c}\left(\left(F_{\xi }^{1},G_{\neg \xi }^{1},N\right)\right)$ .

*Proof*. We prove only (3), (4), and (5), and the remaining are straightforward.

3. By (2.), $\left(F_{\xi }^{2},G_{\neg \xi }^{2},N\right)\tilde{⊑}\overset{˘}{c}\left(\left(F_{\xi }^{2},G_{\neg \xi }^{2},N\right)\right)$. Since $\left(F_{\xi }^{1},G_{\neg \xi }^{1},N\right)\tilde{⊑}\left(F_{\xi }^{2},G_{\neg \xi }^{2},N\right)$, we have $\left(F_{\xi }^{1},G_{\neg \xi }^{1},N\right)\tilde{⊑}\overset{˘}{c}\left(\left(F_{\xi }^{2},G_{\neg \xi }^{2},N\right)\right)$. But $\overset{˘}{c}\left(\left(F_{\xi }^{2},G_{\neg \xi }^{2},N\right)\right)$ is an *N*-BHS closed set. Thus, $\overset{˘}{c}\left(\left(F_{\xi }^{2},G_{\neg \xi }^{2},N\right)\right)$ is an *N*-BHS closed set containing $\left(F_{\xi }^{1},G_{\neg \xi }^{1},N\right)$. Since $\overset{˘}{c}\left(\left(F_{\xi }^{1},G_{\neg \xi }^{1},N\right)\right)$ is the smallest *N*-BHS closed set containing $\left(F_{\xi }^{1},G_{\neg \xi }^{1},N\right)$, so we have $\overset{˘}{c}\left(\left(F_{\xi }^{1},G_{\neg \xi }^{1},N\right)\right)\tilde{⊑}\overset{˘}{c}\left(\left(F_{\xi }^{2},G_{\neg \xi }^{2},N\right)\right)$.
4. Since $\left(F_{\xi }^{1},G_{\neg \xi }^{1},N\right)\tilde{⊑}\left(F_{\xi }^{1},G_{\neg \xi }^{1},N\right)\tilde{⊔}\left(F_{\xi }^{2},G_{\neg \xi }^{2},N\right)$ and $\left(F_{\xi }^{2},G_{\neg \xi }^{2},N\right)\tilde{⊑}\left(F_{\xi }^{1},G_{\neg \xi }^{1},N\right)\tilde{⊔}\left(F_{\xi }^{2},G_{\neg \xi }^{2},N\right)$. By (3.), we have $\overset{˘}{c}\left(\left(F_{\xi }^{1},G_{\neg \xi }^{1},N\right)\right)\tilde{⊑}\overset{˘}{c}\left(\left(F_{\xi }^{1},G_{\neg \xi }^{1},N\right)\;\tilde{⊔}\;\left(F_{\xi }^{2},G_{\neg \xi }^{2},N\right)\right)$ and $\overset{˘}{c}\left(\left(F_{\xi }^{2},G_{\neg \xi }^{2},N\right)\right)\tilde{⊑}\overset{˘}{c}\left(\left(F_{\xi }^{1},G_{\neg \xi }^{1},N\right)\;\tilde{⊔}\;\left(F_{\xi }^{2},G_{\neg \xi }^{2},N\right)\right)$. Hence, $\overset{˘}{c}\left(\left(F_{\xi }^{1},G_{\neg \xi }^{1},N\right)\right)\tilde{⊔}\overset{˘}{c}\left(\left(F_{\xi }^{2},G_{\neg \xi }^{2},N\right)\right)\tilde{⊑}\overset{˘}{c}\left(\left(F_{\xi }^{1},G_{\neg \xi }^{1},N\right)\;\tilde{⊔}\;\left(F_{\xi }^{2},G_{\neg \xi }^{2},N\right)\right)$. Now, since $\overset{˘}{c}\left(\left(F_{\xi }^{1},G_{\neg \xi }^{1},N\right)\right)$ and $\overset{˘}{c}\left(\left(F_{\xi }^{2},G_{\neg \xi }^{2},N\right)\right)$ are *N*-BHS closed sets, $\overset{˘}{c}\left(\left(F_{\xi }^{1},G_{\neg \xi }^{1},N\right)\right)\tilde{⊔}\overset{˘}{c}\left(\left(F_{\xi }^{2},G_{\neg \xi }^{2},N\right)\right)$ is also an *N*-BHS closed. Also, $\left(F_{\xi }^{1},G_{\neg \xi }^{1},N\right)\tilde{⊑}\overset{˘}{c}\left(\left(F_{\xi }^{1},G_{\neg \xi }^{1},N\right)\right)$ and $\left(F_{\xi }^{2},G_{\neg \xi }^{2},N\right)\tilde{⊑}\overset{˘}{c}\left(\left(F_{\xi }^{2},G_{\neg \xi }^{2},N\right)\right)$ implies that $\left(F_{\xi }^{1},G_{\neg \xi }^{1},N\right)\tilde{⊔}\left(F_{\xi }^{2},G_{\neg \xi }^{2},N\right)\tilde{⊑}\overset{˘}{c}\left(\left(F_{\xi }^{1},G_{\neg \xi }^{1},N\right)\right)\tilde{⊔}\overset{˘}{c}\left(\left(F_{\xi }^{2},G_{\neg \xi }^{2},N\right)\right)$. Thus, $\overset{˘}{c}\left(\left(F_{\xi }^{1},G_{\neg \xi }^{1},N\right)\right)\tilde{⊔}\overset{˘}{c}\left(\left(F_{\xi }^{2},G_{\neg \xi }^{2},N\right)\right)$ is an *N*-BHS closed containing $\left(F_{\xi }^{1},G_{\neg \xi }^{1},N\right)\tilde{⊔}\left(F_{\xi }^{2},G_{\neg \xi }^{2},N\right)$. Since $\overset{˘}{c}\left(\left(F_{\xi }^{1},G_{\neg \xi }^{1},N\right)\;\tilde{⊔}\;\left(F_{\xi }^{2},G_{\neg \xi }^{2},N\right)\right)$ is the smallest *N*-BHS closed set containing $\left(F_{\xi }^{1},G_{\neg \xi }^{1},N\right)\tilde{⊔}\left(F_{\xi }^{2},G_{\neg \xi }^{2},N\right)$, we have $\overset{˘}{c}\left(\left(F_{\xi }^{1},G_{\neg \xi }^{1},N\right)\;\tilde{⊔}\;\left(F_{\xi }^{2},G_{\neg \xi }^{2},N\right)\right)\tilde{⊑}\overset{˘}{c}\left(\left(F_{\xi }^{1},G_{\neg \xi }^{1},N\right)\right)\tilde{⊔}\overset{˘}{c}\left(\left(F_{\xi }^{2},G_{\neg \xi }^{2},N\right)\right)$. Hence, $\overset{˘}{c}\left(\left(F_{\xi }^{1},G_{\neg \xi }^{1},N\right)\;\tilde{⊔}\;\left(F_{\xi }^{2},G_{\neg \xi }^{2},N\right)\right)=\overset{˘}{c}\left(\left(F_{\xi }^{1},G_{\neg \xi }^{1},N\right)\right)\tilde{⊔}\overset{˘}{c}\left(\left(F_{\xi }^{2},G_{\neg \xi }^{2},N\right)\right)$.
5. Since $\left(F_{\xi }^{1},G_{\neg \xi }^{1},N\right)\;\tilde{⊓}\;\left(F_{\xi }^{2},G_{\neg \xi }^{2},N\right)\;\tilde{⊑}\;\left(F_{\xi }^{1},G_{\neg \xi }^{1},N\right)$ and $\left(F_{\xi }^{1},G_{\neg \xi }^{1},N\right)\;\tilde{⊓}\left(F_{\xi }^{2},G_{\neg \xi }^{2},N\right)\;\tilde{⊑}\;\left(F_{\xi }^{2},G_{\neg \xi }^{2},N\right)$, then $\overset{˘}{c}\left(\left(F_{\xi }^{1},G_{\neg \xi }^{1},N\right)\;\tilde{⊓}\;\left(F_{\xi }^{2},G_{\neg \xi }^{2},N\right)\right)\tilde{⊑}\overset{˘}{c}\left(\left(F_{\xi }^{1},G_{\neg \xi }^{1},N\right)\right)$ and $\overset{˘}{c}\left(\left(F_{\xi }^{1},G_{\neg \xi }^{1},N\right)\;\tilde{⊓}\;\left(F_{\xi }^{2},G_{\neg \xi }^{2},N\right)\right)\tilde{⊑}\overset{˘}{c}\left(\left(F_{\xi }^{2},G_{\neg \xi }^{2},N\right)\right)$. Therefore, $\overset{˘}{c}\left(\left(F_{\xi }^{1},G_{\neg \xi }^{1},N\right)\;\tilde{⊓}\;\left(F_{\xi }^{2},G_{\neg \xi }^{2},N\right)\right)\tilde{⊑}\overset{˘}{c}\left(\left(F_{\xi }^{1},G_{\neg \xi }^{1},N\right)\right)\tilde{⊓}\overset{˘}{c}\left(\left(F_{\xi }^{2},G_{\neg \xi }^{2},N\right)\right)$.

**Remark 4**. *In general, the equality in Proposition 6 (5) does not hold*.

**Example 4**. *Consider the 6-BHSTS* $\left(¥,{\tilde{ℑ}}_{6},\xi ,6\right)$ *in Example 3 and let* $\left(F_{\xi },G_{\neg \xi },6\right)$ *and* $\left(H_{\xi },K_{\neg \xi },6\right)$ *be any 6-BHS sets defined as follows:*

$\left(F_{\xi },G_{\neg \xi },6\right)$ = {(*ϵ*_1_, {*η*_1_, 0, 5}, {*η*_2_, 1, 2}), (*ϵ*_2_, {*η*_1_, 0, 5}, {*η*_2_, 0, 5})}.

$\left(H_{\xi },K_{\neg \xi },6\right)$ = {(*ϵ*_1_, {*η*_1_, 1, 4}, {*η*_2_, 0, 5}), (*ϵ*_2_, {*η*_1_, 1, 1}, {*η*_2_, 0, 5})}.

*Then*,

$\overset{˘}{c}\left(\left(F_{\xi },G_{\neg \xi },6\right)\right)$ = {(*ϵ*_1_, {*η*_1_, 1, 4}, {*η*_2_, 3, 2}), (*ϵ*_2_, {*η*_1_, 2, 1}, {*η*_2_, 0, 5})}.

*And*,

$\overset{˘}{c}\left(\left(H_{\xi },K_{\neg \xi },6\right)\right)$ = {(*ϵ*_1_, {*η*_1_, 1, 4}, {*η*_2_, 1, 3}), (*ϵ*_2_, {*η*_1_, 2, 1}, {*η*_2_, 0, 5})}.

*Hence*,

$\overset{˘}{c}\left(\left(F_{\xi },G_{\neg \xi },6\right)\right)\tilde{⊓}\overset{˘}{c}\left(\left(H_{\xi },K_{\neg \xi },6\right)\right)$ = {(*ϵ*_1_, {*η*_1_, 1, 4}, {*η*_2_, 1, 3}), (*ϵ*_2_, {*η*_1_, 2, 1}, {*η*_2_, 0, 5})}.

*On the other hand*,

$\left(F_{\xi },G_{\neg \xi },6\right)\tilde{⊓}\left(H_{\xi },K_{\neg \xi },6\right)$ = {(*ϵ*_1_, {*η*_1_, 0, 5}, {*η*_2_, 0, 5}), (*ϵ*_2_, {*η*_1_, 0, 5}, {*η*_2_, 0, 5})} = $\Lambda_{0}^{5}$.

*Then*,

$\overset{˘}{c}\left(\left(F_{\xi },G_{\neg \xi },6\right)\;\tilde{⊓}\;\left(H_{\xi },K_{\neg \xi },6\right)\right)=\overset{˘}{c}\left(\Lambda_{0}^{5}\right)=\Lambda_{0}^{5}$ .

*It follows that* $\overset{˘}{c}\left(\left(F_{\xi },G_{\neg \xi },6\right)\;\tilde{⊓}\;\left(H_{\xi },K_{\neg \xi },6\right)\right)\neq \overset{˘}{c}\left(\left(F_{\xi },G_{\neg \xi },6\right)\right)\tilde{⊓}\overset{˘}{c}\left(\left(H_{\xi },K_{\neg \xi },6\right)\right)$ . *Therefore, in general*, $\overset{˘}{c}\left(\left(F_{\xi },G_{\neg \xi },N\right)\;\tilde{⊓}\;\left(H_{\xi },K_{\neg \xi },N\right)\right)\neq \overset{˘}{c}\left(\left(F_{\xi },G_{\neg \xi },N\right)\right)\tilde{⊓}\overset{˘}{c}\left(\left(H_{\xi },K_{\neg \xi },N\right)\right)$.

**Definition 12**. *Let* $\left(¥,{\tilde{ℑ}}_{ℵ},\xi ,N\right)$ *be an N*-*BHSTS. Then, the N*-*BHS interior of* $\left(F_{\xi },G_{\neg \xi },N\right)$ *is denoted by* $\overset{˘}{i}\left(\left(F_{\xi },G_{\neg \xi },N\right)\right)$ *and is defined as the N*-*BHS union of all N*-*BHS open set contained in* $\left(F_{\xi },G_{\neg \xi },N\right)$.

*In other words*, $\overset{˘}{i}\left(\left(F_{\xi },G_{\neg \xi },N\right)\right)=\tilde{⊔}\{\left(H_{\xi },K_{\neg \xi },N\right)|\left(H_{\xi },K_{\neg \xi },N\right)\in {\tilde{ℑ}}_{ℵ},\left(H_{\xi },K_{\neg \xi },N\right)\tilde{⊑}\left(F_{\xi },G_{\neg \xi },N)\right\}$.

**Remark 5**. *Let* $\left(¥,{\tilde{ℑ}}_{ℵ},\xi ,N\right)$ *be an N*-*BHSTS and* $\left(F_{\xi },G_{\neg \xi },N\right)$ *be an N*-*be an BHS set. Then*,

1.
$\overset{˘}{i}\left(\left(F_{\xi },G_{\neg \xi },N\right)\right)$ *be an is the largest N*-*be an BHS open set contained in* $\left(F_{\xi },G_{\neg \xi },N\right)$.
2.
$\left(F_{\xi },G_{\neg \xi },N\right)$ *be an is an N*-*BHS open set if and only if* $\left(F_{\xi },G_{\neg \xi },N\right)=\overset{˘}{i}\left(\left(F_{\xi },G_{\neg \xi },N\right)\right)$.

**Example 5**. *Consider the 6-BHSTS* $\left(¥,{\tilde{ℑ}}_{6},\xi ,6\right)$ *and the 5-BHS set* $\left(F_{\xi },G_{\neg \xi },6\right)$ *in Example 3. Then*,

$\overset{˘}{i}\left(\left(F_{\xi },G_{\neg \xi },6\right)\right)$ = {(*ϵ*_1_, {*η*_1_, 0, 5}, {*η*_2_, 0, 5}), (*ϵ*_2_, {*η*_1_, 0, 5}, {*η*_2_, 0, 5})} = $\Lambda_{0}^{5}$.

**Proposition 7**. *Let* $\left(¥,{\tilde{ℑ}}_{ℵ},\xi ,N\right)$ *be an N*-*BHSTS and let* $\left(F_{\xi }^{1},G_{\neg \xi }^{1},N\right)$ *and* $\left(F_{\xi }^{2},G_{\neg \xi }^{2},N\right)$ *be two N*-*BHS sets. Then*,

1.
$\overset{˘}{i}\left(\Lambda_{0}^{N-1}\right)=\Lambda_{0}^{N-1}$ *and* $\overset{˘}{i}\left(\Lambda_{N-1}^{0}\right)=\Lambda_{N-1}^{0}$.
2.
$\overset{˘}{i}\left(\left(F_{\xi }^{1},G_{\neg \xi }^{1},N\right)\right)\tilde{⊑}\left(F_{\xi }^{1},G_{\neg \xi }^{1},N\right)$ .
3.
$\left(F_{\xi }^{1},G_{\neg \xi }^{1},N\right)\tilde{⊑}\left(F_{\xi }^{2},G_{\neg \xi }^{2},N\right)$ *implies* $\overset{˘}{i}\left(\left(F_{\xi }^{1},G_{\neg \xi }^{1},N\right)\right)\tilde{⊑}\overset{˘}{i}\left(\left(F_{\xi }^{2},G_{\neg \xi }^{2},N\right)\right)$.
4.
$\overset{˘}{i}(\left(F_{\xi }^{1},G_{\neg \xi }^{1},N\right)\tilde{⊓}\left(F_{\xi }^{2},G_{\neg \xi }^{2},N)\right)=\overset{˘}{i}\left(\left(F_{\xi }^{1},G_{\neg \xi }^{1},N\right)\right)\tilde{⊓}\overset{˘}{i}\left(\left(F_{\xi }^{2},G_{\neg \xi }^{2},N\right)\right)$ .
5.
$\overset{˘}{i}\left(\left(F_{\xi }^{1},G_{\neg \xi }^{1},N\right)\right)\tilde{⊔}\overset{˘}{i}\left(\left(F_{\xi }^{2},G_{\neg \xi }^{2},N\right)\right)\tilde{⊑}\overset{˘}{i}(\left(F_{\xi }^{1},G_{\neg \xi }^{1},N\right)\tilde{⊔}\left(F_{\xi }^{2},G_{\neg \xi }^{2},N)\right)$ .
6.
$\overset{˘}{i}\left(\overset{˘}{i}\left(\left(F_{\xi }^{1},G_{\neg \xi }^{1},N\right)\right)\right)=\overset{˘}{i}\left(\left(F_{\xi }^{1},G_{\neg \xi }^{1},N\right)\right)$ .

*Proof*. We prove only (3), (4), and (5), and the remaining are straightforward.

3. Since $\overset{˘}{i}\left(\left(F_{\xi }^{1},G_{\neg \xi }^{1},N\right)\right)\tilde{⊑}\left(F_{\xi }^{1},G_{\neg \xi }^{1},N\right)$ and $\left(F_{\xi }^{1},G_{\neg \xi }^{1},N\right)\tilde{⊑}\left(F_{\xi }^{2},G_{\neg \xi }^{2},N\right)$, then we have $\overset{˘}{i}\left(\left(F_{\xi }^{1},G_{\neg \xi }^{1},N\right)\right)\tilde{⊑}\left(F_{\xi }^{2},G_{\neg \xi }^{2},N\right)$. But, $\overset{˘}{i}\left(\left(F_{\xi }^{2},G_{\neg \xi }^{2},N\right)\right)$ is the largest *N*-BHS open set contained in $\left(F_{\xi }^{2},G_{\neg \xi }^{2},N\right)$. Thus, $\overset{˘}{i}\left(\left(F_{\xi }^{1},G_{\neg \xi }^{1},N\right)\right)\tilde{⊑}\overset{˘}{i}\left(\left(F_{\xi }^{2},G_{\neg \xi }^{2},N\right)\right)$.
4. Since $\left(F_{\xi }^{1},G_{\neg \xi }^{1},N\right)\tilde{⊓}\left(F_{\xi }^{2},G_{\neg \xi }^{2},N\right)\tilde{⊑}\left(F_{\xi }^{1},G_{\neg \xi }^{1},N\right)$ and $\left(F_{\xi }^{1},G_{\neg \xi }^{1},N\right)\tilde{⊓}\left(F_{\xi }^{2},G_{\neg \xi }^{2},N\right)\tilde{⊑}\left(F_{\xi }^{2},G_{\neg \xi }^{2},N\right)$, then $\overset{˘}{i}(\left(F_{\xi }^{1},G_{\neg \xi }^{1},N\right)\tilde{⊓}\left(F_{\xi }^{2},G_{\neg \xi }^{2},N)\right)\tilde{⊑}\overset{˘}{i}\left(\left(F_{\xi }^{1},G_{\neg \xi }^{1},N\right)\right)$ and $\overset{˘}{i}(\left(F_{\xi }^{1},G_{\neg \xi }^{1},N\right)\tilde{⊓}\left(F_{\xi }^{2},G_{\neg \xi }^{2},N)\right)\tilde{⊑}\overset{˘}{i}\left(\left(F_{\xi }^{2},G_{\neg \xi }^{2},N\right)\right)$. This implies $\overset{˘}{i}(\left(F_{\xi }^{1},G_{\neg \xi }^{1},N\right)\tilde{⊓}\left(F_{\xi }^{2},G_{\neg \xi }^{2},N)\right)\tilde{⊑}\overset{˘}{i}\left(\left(F_{\xi }^{1},G_{\neg \xi }^{1},N\right)\right)\tilde{⊓}\overset{˘}{i}\left(\left(F_{\xi }^{2},G_{\neg \xi }^{2},N\right)\right)$. Now, since $\overset{˘}{i}\left(\left(F_{\xi }^{1},G_{\neg \xi }^{1},N\right)\right)\tilde{⊑}\left(F_{\xi }^{1},G_{\neg \xi }^{1},N\right)$ and $\overset{˘}{i}\left(\left(F_{\xi }^{2},G_{\neg \xi }^{2},N\right)\right)\tilde{⊑}\left(F_{\xi }^{2},G_{\neg \xi }^{2},N\right)$, then $\overset{˘}{i}\left(\left(F_{\xi }^{1},G_{\neg \xi }^{1},N\right)\right)\tilde{⊓}\overset{˘}{i}\left(\left(F_{\xi }^{2},G_{\neg \xi }^{2},N\right)\right)\tilde{⊑}\left(F_{\xi }^{1},G_{\neg \xi }^{1},N\right)\tilde{⊓}\left(F_{\xi }^{2},G_{\neg \xi }^{2},N\right)$. This implies $\overset{˘}{i}(\overset{˘}{i}\left(\left(F_{\xi }^{1},G_{\neg \xi }^{1},N\right)\right)\tilde{⊓}\overset{˘}{i}\left(\left(F_{\xi }^{2},G_{\neg \xi }^{2},N\right)\right))\tilde{⊑}\overset{˘}{i}(\left(F_{\xi }^{1},G_{\neg \xi }^{1},N\right)\tilde{⊓}\left(F_{\xi }^{2},G_{\neg \xi }^{2},N)\right)$. Hence, $\overset{˘}{i}\left(\left(F_{\xi }^{1},G_{\neg \xi }^{1},N\right)\right)\tilde{⊓}\overset{˘}{i}\left(\left(F_{\xi }^{2},G_{\neg \xi }^{2},N\right)\right)\tilde{⊑}\overset{˘}{i}(\left(F_{\xi }^{1},G_{\neg \xi }^{1},N\right)\tilde{⊓}\left(F_{\xi }^{2},G_{\neg \xi }^{2},N)\right)$. Thus, $\overset{˘}{i}\left(\left(F_{\xi }^{1},G_{\neg \xi }^{1},N\right)\right)\tilde{⊓}\overset{˘}{i}\left(\left(F_{\xi }^{2},G_{\neg \xi }^{2},N\right)\right)=\overset{˘}{i}\left(\left(F_{\xi }^{1},G_{\neg \xi }^{1},N\right)\;\tilde{⊓}\;\left(F_{\xi }^{2},G_{\neg \xi }^{2},N\right)\right)$.
5. Since $\left(F_{\xi }^{1},G_{\neg \xi }^{1},N\right)\tilde{⊑}\left(F_{\xi }^{1},G_{\neg \xi }^{1},N\right)\tilde{⊔}\left(F_{\xi }^{2},G_{\neg \xi }^{2},N\right)$, then $\overset{˘}{i}\left(\left(F_{\xi }^{1},G_{\neg \xi }^{1},N\right)\right)\tilde{⊑}\overset{˘}{i}(\left(F_{\xi }^{1},G_{\neg \xi }^{1},N\right)\tilde{⊔}\left(F_{\xi }^{2},G_{\neg \xi }^{2},N)\right)$ and $\left(F_{\xi }^{2},G_{\neg \xi }^{2},N\right)\tilde{⊑}\left(F_{\xi }^{1},G_{\neg \xi }^{1},N\right)\tilde{⊔}\left(F_{\xi }^{2},G_{\neg \xi }^{2},N\right)$, then $\overset{˘}{i}\left(\left(F_{\xi }^{2},G_{\neg \xi }^{2},N\right)\right)\tilde{⊑}\overset{˘}{i}(\left(F_{\xi }^{1},G_{\neg \xi }^{1},N\right)\tilde{⊔}\left(F_{\xi }^{2},G_{\neg \xi }^{2},N)\right)$. This implies $\overset{˘}{i}\left(\left(F_{\xi }^{1},G_{\neg \xi }^{1},N\right)\right)\tilde{⊔}\overset{˘}{i}\left(\left(F_{\xi }^{2},G_{\neg \xi }^{2},N\right)\right)\tilde{⊑}\overset{˘}{i}(\left(F_{\xi }^{1},G_{\neg \xi }^{1},N\right)\tilde{⊔}\left(F_{\xi }^{2},G_{\neg \xi }^{2},N)\right)$.

**Remark 6**. *The next example illustrates that, in general, the equality in Proposition 7 (5) does not hold*.

**Example 6**. *Consider the 6-BHSTS* $\left(¥,{\tilde{ℑ}}_{6},\xi ,6\right)$ *in Example 3 and let* $\left(F_{\xi },G_{\neg \xi },6\right)$ *and* $\left(H_{\xi },K_{\neg \xi },6\right)$ *be any 6-BHS sets defined as follows:*

$\left(F_{\xi },G_{\neg \xi },6\right)$ = {(*ϵ*_1_, {*η*_1_, 5, 0}, {*η*_2_, 4, 0}), (*ϵ*_2_, {*η*_1_, 1, 2}, {*η*_2_, 5, 0})}.

$\left(H_{\xi },K_{\neg \xi },6\right)$ = {(*ϵ*_1_, {*η*_1_, 2, 2}, {*η*_2_, 5, 0}), (*ϵ*_2_, {*η*_1_, 5, 0}, {*η*_2_, 4, 1})}.

*Then*,

$\overset{˘}{i}\left(\left(F_{\xi },G_{\neg \xi },6\right)\right)$ = {(*ϵ*_1_, {*η*_1_, 4, 1}, {*η*_2_, 3, 1}), (*ϵ*_2_, {*η*_1_, 1, 2}, {*η*_2_, 5, 0})} *And*,

$\overset{˘}{i}\left(\left(H_{\xi },K_{\neg \xi },6\right)\right)$ = {(*ϵ*_1_, {*η*_1_, 2, 2}, {*η*_2_, 3, 1}), (*ϵ*_2_, {*η*_1_, 1, 4}, {*η*_2_, 0, 3})}.

*Hence*,

$\overset{˘}{i}\left(\left(F_{\xi },G_{\neg \xi },6\right)\right)\tilde{⊔}\overset{˘}{i}\left(\left(H_{\xi },K_{\neg \xi },5\right)\right)$ {(*ϵ*_1_, {*η*_1_, 4, 1}, {*η*_2_, 3, 1}), (*ϵ*_2_, {*η*_1_, 1, 2}, {*η*_2_, 5, 0})}.

*On the other hand,*

$\left(F_{\xi },G_{\neg \xi },6\right)\tilde{⊔}\left(H_{\xi },K_{\neg \xi },6\right)$ = {(*ϵ*_1_, {*η*_1_, 5, 0}, {*η*_2_, 5, 0}), (*ϵ*_2_, {*η*_1_, 5, 0}, {*η*_2_, 5, 0})} = $\Lambda_{5}^{0}$.

*Then*,

$\overset{˘}{i}\left(\left(F_{\xi },G_{\neg \xi },6\right)\;\tilde{⊔}\;\left(H_{\xi },K_{\neg \xi },6\right)\right)=\overset{˘}{i}\left(\Lambda_{5}^{0}\right)=\Lambda_{5}^{0}$ .

*It follows that* $\overset{˘}{i}\left(\left(F_{\xi },G_{\neg \xi },6\right)\;\tilde{⊔}\;\left(H_{\xi },K_{\neg \xi },6\right)\right)\neq \overset{˘}{i}\left(\left(F_{\xi },G_{\neg \xi },6\right)\right)\tilde{⊔}\overset{˘}{i}\left(\left(H_{\xi },K_{\neg \xi },6\right)\right)$ . *Therefore, in general*, $\overset{˘}{i}\left(\left(F_{\xi },G_{\neg \xi },N\right)\;\tilde{⊔}\;\left(H_{\xi },K_{\neg \xi },N\right)\right)\neq \overset{˘}{i}\left(F_{\xi },G_{\neg \xi },N\right)\tilde{⊔}\overset{˘}{i}\left(\left(H_{\xi },K_{\neg \xi },N\right)\right)$.

**Remark 7**. *Let* $\left(¥,{\tilde{ℑ}}_{ℵ},\xi ,N\right)$ *be an N*-*BHSTS and* $\left(F_{\xi },G_{\neg \xi },N\right)$ *be an N*-*BHS set. Then*, $\overset{˘}{i}\left(F_{\xi },G_{\neg \xi },N\right)\tilde{⊑}\left(F_{\xi },G_{\neg \xi },N\right)\tilde{⊑}\overset{˘}{c}\left(\left(F_{\xi },G_{\neg \xi },N\right)\right)$.

**Proposition 8**. *Let* $\left(¥,{\tilde{ℑ}}_{ℵ},\xi ,N\right)$ *be an N*-*BHSTS and let* $\left(F_{\xi },G_{\neg \xi },N\right)$ *be an N*-*BHS set. Then*,

1.
${\left(\overset{˘}{c}\left(\left(F_{\xi },G_{\neg \xi },N\right)\right)\right)}^{c}=\overset{˘}{i}\left({\left(\left(F_{\xi },G_{\neg \xi },N\right)\right)}^{c}\right)$ .
2.
${\left(\overset{˘}{i}\left(\left(F_{\xi },G_{\neg \xi },N\right)\right)\right)}^{c}=\overset{˘}{c}\left({\left(\left(F_{\xi },G_{\neg \xi },N\right)\right)}^{c}\right)$ .

*Proof*. $\begin{array}{cc} \text{1.}\;\;\;\;\;\;\;\;\;\;\;\;\;\;\overset{˘}{c}\left(\left(F_{\xi },G_{\neg \xi },N\right)\right)\; & =\;\tilde{⊓}\;\left\{\left(H_{\xi },K_{\neg \xi },N\right):{\left(H_{\xi },K_{\neg \xi },N\right)}^{c}\in {\tilde{ℑ}}_{ℵ},\left(H_{\xi },K_{\neg \xi },N\right)\;\tilde{⊒}\;\left(F_{\xi },G_{\neg \xi },N\right)\right\}\\ {\left(\overset{˘}{c}\left(\left(F_{\xi },G_{\neg \xi },N\right)\right)\right)}^{c}\; & =\;{\left(\tilde{⊓}\;\left\{\left(H_{\xi },K_{\neg \xi },N\right):{\left(H_{\xi },K_{\neg \xi },N\right)}^{c}\in {\tilde{ℑ}}_{ℵ},\left(H_{\xi },K_{\neg \xi },N\right)\;\tilde{⊒}\;\left(F_{\xi },G_{\neg \xi },N\right)\right\}\right)}^{c}\\ & =\;\tilde{⊔}\;\left\{{\left(H_{\xi },K_{\neg \xi },N\right)}^{c}:{\left(H_{\xi },K_{\neg \xi },N\right)}^{c}\in {\tilde{ℑ}}_{ℵ},{\left(H_{\xi },K_{\neg \xi },N\right)}^{c}\;\tilde{⊑}\;{\left(F_{\xi },G_{\neg \xi },N\right)}^{c}\right\}\\ & =\;\overset{˘}{i}\left({\left(F_{\xi },G_{\neg \xi },N\right)}^{c}\right). \end{array}$ $\begin{array}{cc} \text{2.}\;\;\;\;\;\;\;\;\;\;\;\;\;\;\overset{˘}{i}\left(\left(F_{\xi },G_{\neg \xi },N\right)\right)\; & =\;\tilde{⊔}\;\left\{\left(H_{\xi },K_{\neg \xi },N\right):\left(H_{\xi },K_{\neg \xi },N\right)\in {\tilde{ℑ}}_{ℵ},\left(H_{\xi },K_{\neg \xi },N\right)\;\tilde{⊑}\;\left(F_{\xi },G_{\neg \xi },N\right)\right\}\\ {\left(\overset{˘}{i}\left(\left(F_{\xi },G_{\neg \xi },N\right)\right)\right)}^{c}\; & =\;{\left(\tilde{⊔}\;\left\{\left(H_{\xi },K_{\neg \xi },N\right):\left(H_{\xi },K_{\neg \xi },N\right)\in {\tilde{ℑ}}_{ℵ},\left(H_{\xi },K_{\neg \xi },N\right)\;\tilde{⊑}\;\left(F_{\xi },G_{\neg \xi },N\right)\right\}\right)}^{c}\\ & =\;\tilde{⊓}\;\left\{{\left(H_{\xi },K_{\neg \xi },N\right)}^{c}:\left(H_{\xi },K_{\neg \xi },N\right)\in {\tilde{ℑ}}_{ℵ},{\left(F_{\xi },G_{\neg \xi },N\right)}^{c}\;\tilde{⊑}\;{\left(\left(H_{\xi },K_{\neg \xi },N\right)\right)}^{c}\right\}\\ & =\;\overset{˘}{c}\left({\left(F_{\xi },G_{\neg \xi },N\right)}^{c}\right). \end{array}$

**Definition 13**. *Let* $\left(¥,{\tilde{ℑ}}_{ℵ},\xi ,N\right)$ *be an N*-*BHSTS and* $\left(F_{\xi },G_{\neg \xi },N\right)$ *be an N*-*BHS set. Then, the N*-*BHS exterior of* $\left(F_{\xi },G_{\neg \xi },N\right)$ *is denoted and defined as follows:*

$\overset{˘}{e}\left(\left(F_{\xi },G_{\neg \xi },N\right)\right)=\tilde{⊔}\{\left(H_{\xi },K_{\neg \xi },N\right)|\left(H_{\xi },K_{\neg \xi },N\right)\in {\tilde{ℑ}}_{ℵ},\left(H_{\xi },K_{\neg \xi },N\right)\tilde{⊑}{\left(F_{\xi },G_{\neg \xi },N\right)}^{c}\}$ .

**Example 7**. *Consider the 6-BHSTS* $\left(¥,{\tilde{ℑ}}_{6},\xi ,6\right)$ *and the 6-BHS set* $\left(F_{\xi },G_{\neg \xi },6\right)$ *in Example 3. Then*,

$\overset{˘}{e}\left(\left(F_{\xi },G_{\neg \xi },6\right)\right)$ = {(*ϵ*_1_, {*η*_1_, 4, 1}, {*η*_2_, 2, 3}), (*ϵ*_2_, {*η*_1_, 1, 2}, {*η*_2_, 5, 0})}.

**Proposition 9**. *Let* $\left(¥,{\tilde{ℑ}}_{ℵ},\xi ,N\right)$ *be an N*-*BHSTS and let* $\left(F_{\xi }^{1},G_{\neg \xi }^{1},N\right)$ *and* $\left(F_{\xi }^{2},G_{\neg \xi }^{2},N\right)$ *be two N*-*BHS sets. Then*,

1.
$\overset{˘}{e}\left(\Lambda_{N-1}^{0}\right)=\Lambda_{0}^{N-1}$ *and* $\overset{˘}{e}\left(\Lambda_{0}^{N-1}\right)=\Lambda_{N-1}^{0}$ .
2.
$\left(F_{\xi }^{1},G_{\neg \xi }^{1},N\right)\tilde{⊒}\left(F_{\xi }^{2},G_{\neg \xi }^{2},N\right)$ *implies* $\overset{˘}{e}\left(\left(F_{\xi }^{2},G_{\neg \xi }^{2},N\right)\right)\tilde{⊑}\overset{˘}{e}\left(\left(F_{\xi }^{1},G_{\neg \xi }^{1},N\right)\right)$ .
3.
$\overset{˘}{e}(\left(F_{\xi }^{1},G_{\neg \xi }^{1},N\right)\tilde{⊔}\left(F_{\xi }^{2},G_{\neg \xi }^{2},N)\right)=\overset{˘}{e}\left(\left(F_{\xi }^{1},G_{\neg \xi }^{1},N\right)\right)\tilde{⊓}\overset{˘}{e}\left(\left(F_{\xi }^{2},G_{\neg \xi }^{2},N\right)\right)$ .
4.
$\overset{˘}{e}(\left(F_{\xi }^{1},G_{\neg \xi }^{1},N\right)\tilde{⊓}\left(F_{\xi }^{2},G_{\neg \xi }^{2},N)\right)\tilde{⊑}\overset{˘}{e}\left(\left(F_{\xi }^{1},G_{\neg \xi }^{1},N\right)\right)\tilde{⊔}\overset{˘}{e}\left(\left(F_{\xi }^{2},G_{\neg \xi }^{2},N\right)\right)$ .

*Proof*.

1.
$\overset{˘}{e}\left(\Lambda_{N-1}^{0}\right)=\overset{˘}{i}\left({\left(\Lambda_{N-1}^{0}\right)}^{c}\right)=\overset{˘}{i}\left(\Lambda_{0}^{N-1}\right)=\Lambda_{0}^{N-1}$ .

$\overset{˘}{e}\left(\Lambda_{0}^{N-1}\right)=\overset{˘}{i}\left({\left(\Lambda_{0}^{N-1}\right)}^{c}\right)=\overset{˘}{i}\left(\Lambda_{N-1}^{0}\right)=\Lambda_{N-1}^{0}$ .
2.
$\left(F_{\xi }^{1},G_{\neg \xi }^{1},N\right)\tilde{⊑}\left(F_{\xi }^{2},G_{\neg \xi }^{2},N\right)$ then ${\left(F_{\xi }^{2},G_{\neg \xi }^{2},N\right)}^{c}\tilde{⊑}{\left(F_{\xi }^{1},G_{\neg \xi }^{1},N\right)}^{c}$. This implies $\overset{˘}{i}\left({\left(F_{\xi }^{2},G_{\neg \xi }^{2},N\right)}^{c}\right)\tilde{⊑}\overset{˘}{i}\left({\left(F_{\xi }^{1},G_{\neg \xi }^{1},N\right)}^{c}\right)$. Hence, $\overset{˘}{e}\left(\left(F_{\xi }^{2},G_{\neg \xi }^{2},N\right)\right)\tilde{⊑}\overset{˘}{e}\left(\left(F_{\xi }^{1},G_{\neg \xi }^{1},N\right)\right)$.
3.
$\overset{˘}{e}(\left(F_{\xi }^{1},G_{\neg \xi }^{1},N\right)\tilde{⊔}\left(F_{\xi }^{2},G_{\neg \xi }^{2},N)\right)=\overset{˘}{i}((\left(F_{\xi }^{1},G_{\neg \xi }^{1},N\right)\tilde{⊔}\left(F_{\xi }^{2},G_{\neg \xi }^{2},N\right){)}^{c})=\overset{˘}{i}({\left(F_{\xi }^{1},G_{\neg \xi }^{1},N\right)}^{c}\tilde{⊓}{\left(F_{\xi }^{2},G_{\neg \xi }^{2},N\right)}^{c})=\overset{˘}{i}\left({\left(F_{\xi }^{1},G_{\neg \xi }^{1},N\right)}^{c}\right)\tilde{⊓}\overset{˘}{i}\left({\left(F_{\xi }^{2},G_{\neg \xi }^{2},N\right)}^{c}\right)=\overset{˘}{e}\left(\left(F_{\xi }^{1},G_{\neg \xi }^{1},N\right)\right)\tilde{⊓}\overset{˘}{e}\left(\left(F_{\xi }^{2},G_{\neg \xi }^{2},N\right)\right)$ .
4.
$\overset{˘}{e}(\left(F_{\xi }^{1},G_{\neg \xi }^{1},N\right)\tilde{⊓}\left(F_{\xi }^{2},G_{\neg \xi }^{2},N)\right)=\overset{˘}{i}((\left(F_{\xi }^{1},G_{\neg \xi }^{1},N\right)\tilde{⊓}\left(F_{\xi }^{2},G_{\neg \xi }^{2},N\right){)}^{c})=\overset{˘}{i}({\left(F_{\xi }^{1},G_{\neg \xi }^{1},N\right)}^{c}\tilde{⊔}{\left(F_{\xi }^{2},G_{\neg \xi }^{2},N\right)}^{c})\tilde{⊒}\overset{˘}{i}\left({\left(F_{\xi }^{1},G_{\neg \xi }^{1},N\right)}^{c}\right)\tilde{⊔}\overset{˘}{i}\left({\left(F_{\xi }^{2},G_{\neg \xi }^{2},N\right)}^{c}\right)=\overset{˘}{e}\left(\left(F_{\xi }^{1},G_{\neg \xi }^{1},N\right)\right)\tilde{⊔}\overset{˘}{e}\left(\left(F_{\xi }^{2},G_{\neg \xi }^{2},N\right)\right)$ .

**Definition 14**. *Let* $\left(¥,{\tilde{ℑ}}_{ℵ},\xi ,N\right)$ *be an N*-*BHSTS. Then, N*-*BHS boundary of* $\left(F_{\xi },G_{\neg \xi },N\right)$ *is denoted by* $\overset{˘}{b}\left(\left(F_{\xi },G_{\neg \xi },N\right)\right)$ *and is defined as* $\overset{˘}{b}\left(F_{\xi },G_{\neg \xi },N\right)=\overset{˘}{c}\left(\left(F_{\xi },G_{\neg \xi },N\right)\right)\tilde{⊓}\overset{˘}{c}\left({\left(F_{\xi },G_{\neg \xi },N\right)}^{c}\right)$.

**Remark 8**. *According to Definition 14, it follows that the N*-*BHS sets* $\left(F_{\xi },G_{\neg \xi },N\right)$ *and* ${\left(F_{\xi },G_{\neg \xi },N\right)}^{c}$ *share the same N*-*BHS boundary*.

**Example 8**. *Consider the 6-BHSTS* $\left(¥,{\tilde{ℑ}}_{6},\xi ,6\right)$ *and the 6-BHS set* $\left(F_{\xi },G_{\neg \xi },6\right)$ *in Example 3. Then*,

$\overset{˘}{b}\left(\left(F_{\xi },G_{\neg \xi },6\right)\right)$ = {(*ϵ*_1_, {*η*_1_, 1, 4}, {*η*_2_, 3, 2}), (*ϵ*_2_, {*η*_1_, 2, 1}, {*η*_2_, 0, 5})}.

**Proposition 10**. *Let* $\left(¥,{\tilde{ℑ}}_{ℵ},\xi ,N\right)$ *be an N-BHSTS and let* $\left(F_{\xi },G_{\neg \xi },N\right)$ *be an N*-*BHS set. Then*,

1.
$\overset{˘}{b}\left(\left(F_{\xi },G_{\neg \xi },N\right)\right)\tilde{⊑}\overset{˘}{c}\left(\left(F_{\xi },G_{\neg \xi },N\right)\right)$ .
2.
${\left(\overset{˘}{b}\left(\left(F_{\xi },G_{\neg \xi },N\right)\right)\right)}^{c}=\overset{˘}{i}\left(\left(F_{\xi },G_{\neg \xi },N\right)\right)\tilde{⊔}\overset{˘}{e}\left(\left(F_{\xi },G_{\neg \xi },N\right)\right)$ .
3.
$\overset{˘}{b}\left(\overset{˘}{i}\left(\left(F_{\xi },G_{\neg \xi },N\right)\right)\right)\tilde{⊑}\overset{˘}{b}\left(\left(F_{\xi },G_{\neg \xi },N\right)\right)$ .
4.
$\overset{˘}{b}\left(\overset{˘}{c}\left(\left(F_{\xi },G_{\neg \xi },N\right)\right)\right)\tilde{⊑}\overset{˘}{b}\left(\left(F_{\xi },G_{\neg \xi },N\right)\right)$ .

*Proof*.

1.
$\overset{˘}{b}\left(\left(F_{\xi },G_{\neg \xi },N\right)\right)=\overset{˘}{c}\left(\left(F_{\xi },G_{\neg \xi },N\right)\right)\tilde{⊓}\overset{˘}{c}\left({\left(F_{\xi },G_{\neg \xi },N\right)}^{c}\right)$ . Hence, $\overset{˘}{b}\left(\left(F_{\xi },G_{\neg \xi },N\right)\right)\tilde{⊑}\overset{˘}{c}\left(\left(F_{\xi },G_{\neg \xi },N\right)\right)$.
2.
${\left(\overset{˘}{b}\left(\left(F_{\xi },G_{\neg \xi },N\right)\right)\right)}^{c}=[\overset{˘}{c}\left(\left(F_{\xi },G_{\neg \xi },N\right)\right)\tilde{⊓}\overset{˘}{c}\left({\left(F_{\xi },G_{\neg \xi },N\right)}^{c}\right){]}^{c}={\left(\overset{˘}{c}\left(\left(F_{\xi },G_{\neg \xi },N\right)\right)\right)}^{c}\tilde{⊔}{\left(\overset{˘}{c}\left({\left(F_{\xi },G_{\neg \xi },N\right)}^{c}\right)\right)}^{c}=\overset{˘}{i}\left({\left(F_{\xi },G_{\neg \xi },N\right)}^{c}\right)\tilde{⊔}\overset{˘}{i}\left(\left(F_{\xi },G_{\neg \xi },N\right)\right)=\overset{˘}{e}\left(\left(F_{\xi },G_{\neg \xi },N\right)\right)\tilde{⊔}\overset{˘}{i}\left(\left(F_{\xi },G_{\neg \xi },N\right)\right)$ .
3.
$\overset{˘}{b}\left(\overset{˘}{i}\left(\left(F_{\xi },G_{\neg \xi },N\right)\right)\right)=\overset{˘}{c}\left(\overset{˘}{i}\left(\left(F_{\xi },G_{\neg \xi },N\right)\right)\right)\tilde{⊓}\overset{˘}{c}\left({\left(\overset{˘}{i}\left(\left(F_{\xi },G_{\neg \xi },N\right)\right)\right)}^{c}\right)=\overset{˘}{c}\left(\overset{˘}{i}\left(\left(F_{\xi },G_{\neg \xi },N\right)\right)\right)\tilde{⊓}\overset{˘}{c}\left(\overset{˘}{c}\left({\left(F_{\xi },G_{\neg \xi },N\right)}^{c}\right)\right)\tilde{⊑}\overset{˘}{c}\left(\left(F_{\xi },G_{\neg \xi },N\right)\right)\tilde{⊓}\overset{˘}{c}\left({\left(F_{\xi },G_{\neg \xi },N\right)}^{c}\right)=\overset{˘}{b}\left(\left(F_{\xi },G_{\neg \xi },N\right)\right)$ .
4.
$\overset{˘}{b}\left(\overset{˘}{c}\left(\left(F_{\xi },G_{\neg \xi },N\right)\right)\right)=\overset{˘}{c}\left(\overset{˘}{c}\left(\left(F_{\xi },G_{\neg \xi },N\right)\right)\right)\tilde{⊓}\overset{˘}{c}\left({\left(\overset{˘}{c}\left(\left(F_{\xi },G_{\neg \xi },N\right)\right)\right)}^{c}\right)\tilde{⊑}\overset{˘}{c}\left(\left(F_{\xi },G_{\neg \xi },N\right)\right)\tilde{⊓}\overset{˘}{c}\left({\left(F_{\xi },G_{\neg \xi },N\right)}^{c}\right)=\overset{˘}{b}\left(\left(F_{\xi },G_{\neg \xi },N\right)\right)$ .

**Proposition 11**. *Let* $\left(¥,{\tilde{ℑ}}_{ℵ},\xi ,N\right)$ *be an N*-*BHSTS and let* $\left(F_{\xi },G_{\neg \xi },N\right)$ *be an N*-*BHS set. Then*,

1.
$\overset{˘}{i}\left(\left(F_{\xi },G_{\neg \xi },N\right)\right)\tilde{⊔}\overset{˘}{b}\left(\left(F_{\xi },G_{\neg \xi },N\right)\right)\tilde{⊑}\overset{˘}{c}\left(\left(F_{\xi },G_{\neg \xi },N\right)\right)$ .
2.
$\overset{˘}{i}\left(\left(F_{\xi },G_{\neg \xi },N\right)\right)\tilde{⊔}\overset{˘}{e}\left(\left(F_{\xi },G_{\neg \xi },N\right)\right)\tilde{⊔}\overset{˘}{b}\left(\left(F_{\xi },G_{\neg \xi },N\right)\right)\tilde{⊑}\Lambda_{N-1}^{0}$ .

*Proof*.

1.
$\overset{˘}{i}\left(\left(F_{\xi },G_{\neg \xi },N\right)\right)\tilde{⊔}\overset{˘}{b}\left(\left(F_{\xi },G_{\neg \xi },N\right)\right)=\overset{˘}{i}\left(\left(F_{\xi },G_{\neg \xi },N\right)\right)\tilde{⊔}[\overset{˘}{c}\left(\left(F_{\xi },G_{\neg \xi },N\right)\right)\tilde{⊓}\overset{˘}{c}\left({\left(F_{\xi },G_{\neg \xi },N\right)}^{c}\right)]=[\overset{˘}{i}\left(\left(F_{\xi },G_{\neg \xi },N\right)\right)\tilde{⊔}\overset{˘}{c}\left(\left(F_{\xi },G_{\neg \xi },N\right)\right)]\tilde{⊓}[\overset{˘}{i}\left(\left(F_{\xi },G_{\neg \xi },N\right)\right)\tilde{⊔}\overset{˘}{c}\left({\left(F_{\xi },G_{\neg \xi },N\right)}^{c}\right)]=\overset{˘}{c}\left(\left(F_{\xi },G_{\neg \xi },N\right)\right)\tilde{⊓}[\overset{˘}{i}\left(\left(F_{\xi },G_{\neg \xi },N\right)\right)\tilde{⊔}{\left(\overset{˘}{i}\left(\left(F_{\xi },G_{\neg \xi },N\right)\right)\right)}^{c}]\tilde{⊑}\overset{˘}{c}\left(\left(F_{\xi },G_{\neg \xi },N\right)\right)\tilde{⊓}\Lambda_{N-1}^{0}=\overset{˘}{c}\left(\left(F_{\xi },G_{\neg \xi },N\right)\right)$ .
2. By Proposition 10 (2), $\overset{˘}{i}\left(\left(F_{\xi },G_{\neg \xi },N\right)\right)\tilde{⊔}\overset{˘}{e}\left(\left(F_{\xi },G_{\neg \xi },N\right)\right)={\left(\overset{˘}{b}\left(\left(F_{\xi },G_{\neg \xi },N\right)\right)\right)}^{c}$, then $\overset{˘}{i}\left(\left(F_{\xi },G_{\neg \xi },N\right)\right)\tilde{⊔}\overset{˘}{e}\left(\left(F_{\xi },G_{\neg \xi },N\right)\right)\tilde{⊔}\overset{˘}{b}\left(\left(F_{\xi },G_{\neg \xi },N\right)\right)={\left(\overset{˘}{b}\left(\left(F_{\xi },G_{\neg \xi },N\right)\right)\right)}^{c}\tilde{⊔}\overset{˘}{b}\left(\left(F_{\xi },G_{\neg \xi },N\right)\right)\tilde{⊑}\Lambda_{N-1}^{0}$.

## 5 Advancements in MCGDM through *N*-bipolar hypersoft topology

In this section, we delve into the advancements made in MCGDM using *N*-BHST. Our methodology sets itself apart from existing literature by harnessing ${\tilde{ℑ}}_{ℵ}$ to incorporate a spectrum of opinions, encompassing complete disagreement $\Lambda_{0}^{N-1}$, complete agreement $\Lambda_{N-1}^{0}$, and expertise in partial agreement levels denoted as $\left(F_{\xi }^{k},G_{\neg \xi }^{k},N\right)$ for *k* = 1, 2, …, *p*.

Two distinct algorithms, namely Algorithm 1 and Algorithm 2, are introduced to augment the MCGDM process through *N*-BHST. Notably, despite their unique methodologies, both algorithms converge towards the same optimal choice. The rankings generated by these algorithms exhibit remarkable similarity, underscoring the robust nature of our approach and its ability to provide a consistent and reliable decision-making outcome. Further sections will unravel the complexities of these algorithms, complemented by a numerical example that illustrates their practical application in real-world decision scenarios.

**Algorithm 1.** *N*-BHST-Aggregate Rank.

1. Input:
  (a) ¥ = {*η*_*i*_, *i* = 1, 2, …, *m*} as a set of alternatives.
  (b) *ξ* = {*ϵ*_*j*_, *j* = 1, 2, …, *n*} as a set of attributes.
  (c) ${\tilde{ℑ}}_{ℵ}=\left\{\Lambda_{0}^{N-1},\Lambda_{N-1}^{0},\left(F_{\xi }^{k},G_{\neg \xi }^{k},N\right)|k=1,2,...,p\right\}$ as an *N*-BHST.
2. Calculations:
  (a) For each *N*-BHS open set $\left(F_{\xi }^{k},G_{\neg \xi }^{k},N\right)$, compute the aggregate *N*-BHS set:
    $$\begin{array}{c} {\left(F_{\xi }^{k},G_{\neg \xi }^{k},N\right)}^{*}=\{\frac{{\left(F_{\xi }^{k},N\right)}^{*}-{\left(G_{\neg \xi }^{k},N\right)}^{*}}{\eta_{i}}|\eta_{i}\in ¥\}, \end{array}$$
    where
    $$\begin{array}{c} {\left(F_{\xi }^{k},N\right)}^{*}=\sum_{j=1}^{n}r_{ij}^{+}, \end{array}$$
    and
    $$\begin{array}{c} {\left(G_{\neg \xi }^{k},N\right)}^{*}=\sum_{j=1}^{n}r_{ij}^{-}. \end{array}$$
  (b) For each alternative *η*_*i*_, compute the aggregate value as the sum of the aggregate values across all *N*-BHS open sets:
    $$\begin{array}{c} {\text{Agg}}_{\eta_{i}}=\sum_{k}{\left(F_{\xi }^{k},G_{\neg \xi }^{k},N\right)}^{*}. \end{array}$$
3. Output: Choose the optimal choice *η* as the alternative with the maximum aggregate value:
  $$\begin{array}{c} \eta =\underset{i}{\text{max}}\left\{{\text{Agg}}_{\eta_{i}}\right\}. \end{array}$$

**Fig 1:**
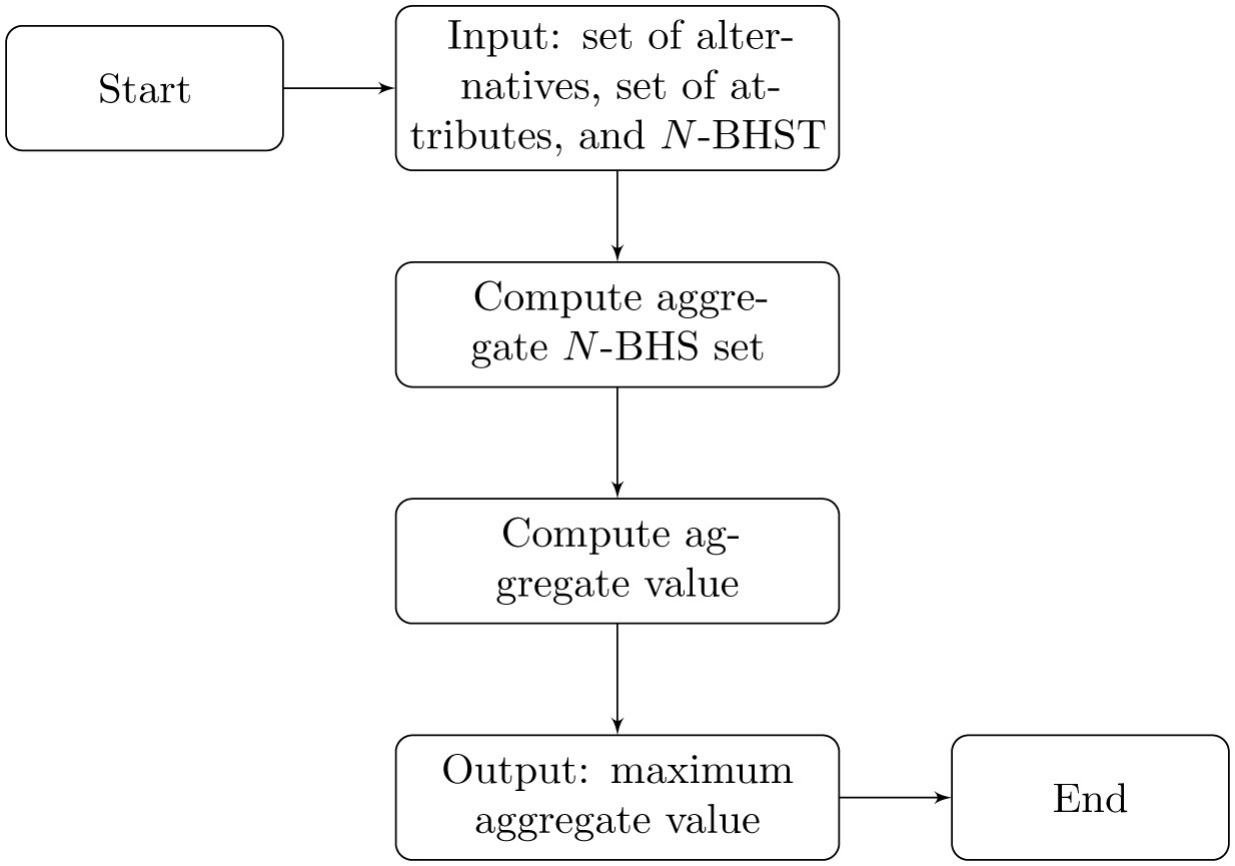
Flowchart for Algorithm 1.

The flowchart of Algorithm 1 is given in Fig 1.

**Algorithm 2.** *N*-BHST-Cardinal Rank.

1. Input:
  (a) ¥ = {*η*_*i*_, *i* = 1, 2, …, *m*} as a set of alternatives.
  (b) *ξ* = {*ϵ*_*j*_, *j* = 1, 2, …, *n*} as a set of attributes.
  (c) ${\tilde{ℑ}}_{ℵ}=\left\{\Lambda_{0}^{N-1},\Lambda_{N-1}^{0},\left(F_{\xi }^{k},G_{\neg \xi }^{k},N\right)|k=1,2,...,p\right\}$ as an *N*-BHST.
2. Calculations:
  (a) For each *N*-BHS open set $\left(F_{\xi }^{k},G_{\neg \xi }^{k},N\right)$, compute the cardinal *N*-HS set:
    $$\begin{array}{c} {\left(F_{\xi }^{k},N\right)}^{•}=\{\frac{\sum_{i=1}^{n}r_{ij}^{+}}{\epsilon_{j}}|\epsilon_{j}\in \xi \}. \end{array}$$
    $$\begin{array}{c} {\left(G_{\neg \xi }^{k},N\right)}^{•}=\{\frac{\sum_{i=1}^{n}r_{ij}^{-}}{\epsilon_{j}}|\epsilon_{j}\in \xi \}. \end{array}$$
  (b) Find the aggregate *N*-HS set by using the formula:
    $$\begin{array}{c} M_{{\left(F_{\xi }^{k},N\right)}^{*}}=M_{\left(F_{\xi }^{k},N\right)}\times M_{{\left(F_{\xi }^{k},N\right)}^{•}}^{T}, \end{array}$$
    $$\begin{array}{c} M_{{\left(G_{\neg \xi }^{k},N\right)}^{*}}=M_{\left(G_{\neg \xi }^{k},N\right)}\times M_{{\left(G_{\neg \xi }^{k},N\right)}^{•}}^{T}, \end{array}$$
    where $M_{{\left(F_{\xi }^{k},N\right)}^{*}},M_{\left(F_{\xi }^{k},N\right)}$, and $M_{{\left(F_{\xi }^{k},N\right)}^{•}}^{T}$ are matrices corresponding to ${\left(F_{\xi }^{k},N\right)}^{*},\left(F_{\xi }^{k},N\right)$, and ${\left(F_{\xi }^{k},N\right)}^{•}$, respectively. The matrix $M_{{\left(F_{\xi }^{k},N\right)}^{•}}^{T}$ represents the transpose of the matrix $M_{{\left(F_{\xi }^{k},N\right)}^{•}}$.
  (c) Find the aggregate *N*-BHS set for each *N*-BHS open set by using the formula:
    $$\begin{array}{c} {\left(F_{\xi }^{k},G_{\neg \xi }^{k},N\right)}^{*}={\left(F_{\xi }^{k},N\right)}^{*}-{\left(G_{\neg \xi }^{k},N\right)}^{*}. \end{array}$$
  (d) For each alternative *η*_*i*_, compute the aggregate value as the sum of the aggregate values across all *N*-BHS open sets:
    $$\begin{array}{c} {\text{Agg}}_{\eta_{i}}=\sum_{k}{\left(F_{\xi }^{k},G_{\neg \xi }^{k},N\right)}^{*}. \end{array}$$
3. Output: Choose the optimal choice *η* as the alternative with the maximum aggregate value:
  $$\begin{array}{c} \eta =\underset{i}{\text{max}}\left\{{\text{Agg}}_{\eta_{i}}\right\}. \end{array}$$

**Fig 2:**
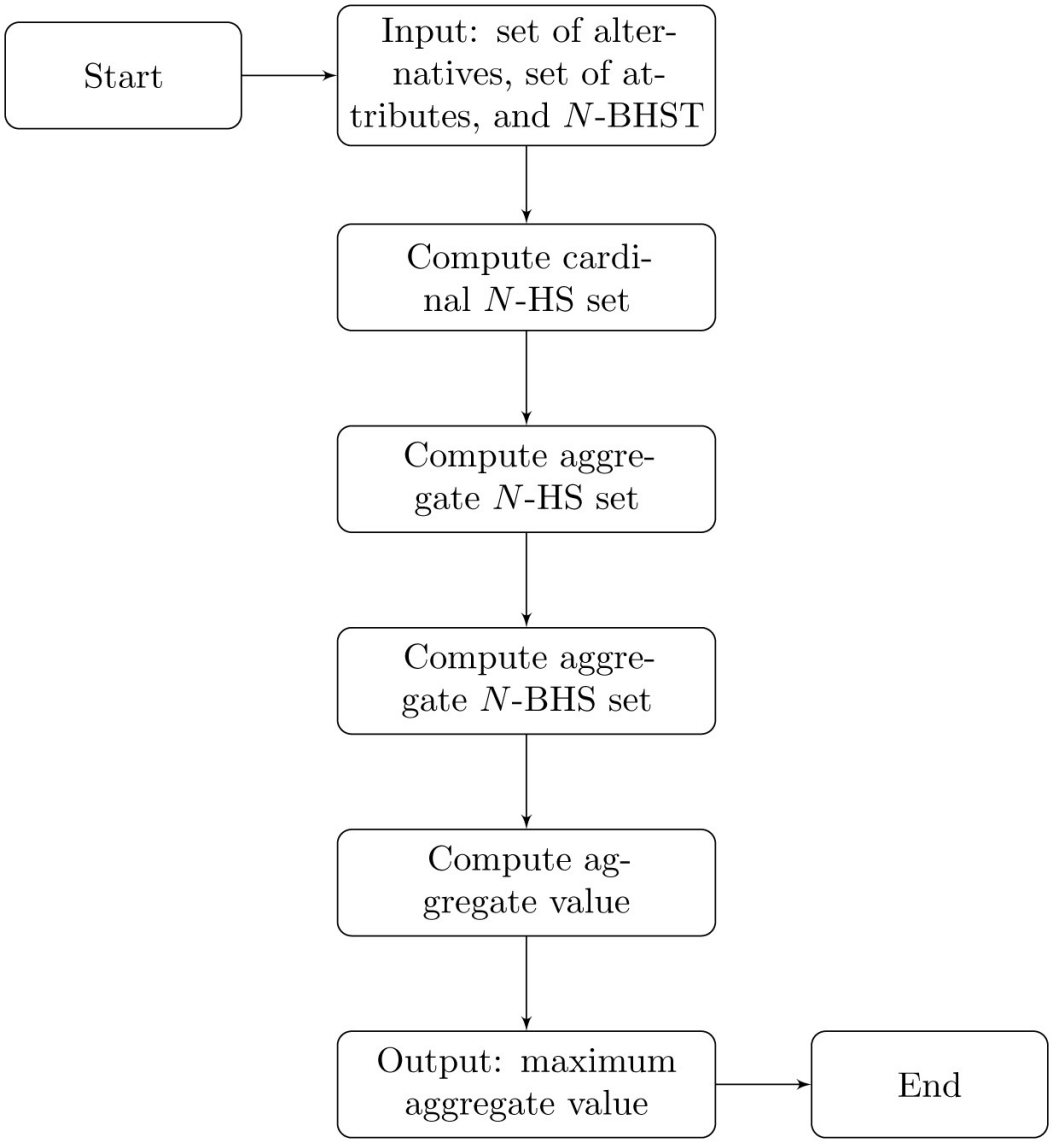
Flowchart for Algorithm 2.

The flowchart of Algorithm 2 is given in Fig 2.

### Numerical example: Selecting a project manager

Suppose a project team is tasked with selecting a project manager from a pool of candidates, denoted as ¥ = {*η*_1_,*η*_2_, *η*_3_, *η*_4_, *η*_5_}. The selection process involves evaluating candidates based on three critical attributes: Leadership Skills, Technical Expertise, and Communication Skills. These attributes are further broken down into sub-attributes, forming the set of all possible skills *ξ* = *ξ*_1_ × *ξ*_2_ × *ξ*_3_, where:

- *ξ*_1_ = Leadership Skills with sub-attributes *e*_1_ = Team Management, *e*_2_ = Decision- Making, *e*_3_ = Conflict Resolution,
- *ξ*_2_ = Technical Expertise with sub-attributes *e*_4_ = Project Planning,
- *ξ*_3_ = Communication Skills with sub-attributes *e*_5_ = Written Communication.

The ordered grades *R* = {0, 1, 2} are used to assess candidates, representing Novice to Master levels. A 3-BHST ${\tilde{ℑ}}_{3}$ is employed to capture different levels of agreement and disagreement. The expertise assessments for complete disagreement $\Lambda_{0}^{2}$, complete agreement $\Lambda_{2}^{0}$, and two partial agreement scenarios $\left(F_{\xi }^{1},G_{\neg \xi }^{1},3\right)$ and $\left(F_{\xi }^{2},G_{\neg \xi }^{2},3\right)$ are provided in Tables 3 and 4.

**Table 3:** Tabular representation of *N*-BHS open set $\left(F_{\xi }^{1},G_{\neg \xi }^{1},3\right)$.

$\left(F_{\xi }^{1},G_{\neg \xi }^{1},3\right)$	*ϵ*_1_	*ϵ*_2_	*ϵ*_3_
*η*_1_	(2,1)	(0,1)	(2,0)
*η*_2_	(2,0)	(1,1)	(1,0)
*η*_3_	(1,0)	(0,0)	(0,0)
*η*_4_	(1,1)	(0,2)	(0,2)
*η*_5_	(1,1)	(2,0)	(0,2)
*η*_6_	(1,0)	(2,0)	(1,0)
*η*_7_	(1,0)	(2,0)	(0,0)

**Table 4:** Tabular representation of *N*-BHS open set $\left(F_{\xi }^{2},G_{\neg \xi }^{2},3\right)$.

$\left(F_{\xi }^{2},G_{\neg \xi }^{2},3\right)$	*ϵ*_1_	*ϵ*_2_	*ϵ*_3_
*η*_1_	(1,1)	(0,2)	(0,0)
*η*_2_	(1,0)	(1,1)	(1,0)
*η*_3_	(0,1)	(0,0)	(0,1)
*η*_4_	(1,1)	(0,2)	(0,2)
*η*_5_	(1,1)	(0,0)	(0,2)
*η*_6_	(0,1)	(0,2)	(1,1)
*η*_7_	(1,1)	(1,0)	(0,2)

We employ both Algorithm 1 and Algorithm 1 to determine the most suitable candidate for the project manager position. It’s important to highlight that, in the computations, we intentionally exclude the *N*-BHS open sets $\Lambda_{0}^{2}$ and $\Lambda_{2}^{0}$ as their inclusion does not influence the ranking.

#### 5.1 Approach 1: Utilizing Algorithm 1 for project manager selection

In this methodology, Algorithm 1 is utilized to enhance the MCGDM process with the integration of *N*-BHST. The specific steps involved are outlined below, applying them to a practical example of selecting a project manager.

1. Compute the aggregate 3-BHS set for each 3-BHS open set:
  $$\begin{array}{cc} {\left(F_{\xi }^{1},G_{\neg \xi }^{1},3\right)}^{*} & =\{\frac{2}{\eta_{1}},\frac{3}{\eta_{2}},\frac{1}{\eta_{3}},\frac{-5}{\eta_{4}},\frac{0}{\eta_{5}},\frac{4}{\eta_{6}},\frac{3}{\eta_{7}}\}.\\ {\left(F_{\xi }^{2},G_{\neg \xi }^{2},3\right)}^{*} & =\{\frac{-2}{\eta_{1}},\frac{2}{\eta_{2}},\frac{-2}{\eta_{3}},\frac{-4}{\eta_{4}},\frac{-2}{\eta_{5}},\frac{-3}{\eta_{6}},\frac{-1}{\eta_{7}}\}. \end{array}$$
2. Compute the aggregate value for each alternative:
  $$\begin{array}{cc} {\text{Agg}}_{\eta_{1}} & =\frac{2}{\eta_{1}}+\frac{-2}{\eta_{1}}=\frac{0}{\eta_{1}}.\\ {\text{Agg}}_{\eta_{2}} & =\frac{3}{\eta_{2}}+\frac{2}{\eta_{2}}=\frac{5}{\eta_{2}}.\\ {\text{Agg}}_{\eta_{3}} & =\frac{1}{\eta_{3}}+\frac{-2}{\eta_{3}}=\frac{-1}{\eta_{3}}.\\ {\text{Agg}}_{\eta_{4}} & =\frac{-5}{\eta_{4}}+\frac{-4}{\eta_{4}}=\frac{-9}{\eta_{4}}.\\ {\text{Agg}}_{\eta_{5}} & =\frac{0}{\eta_{5}}+\frac{-2}{\eta_{5}}=\frac{-2}{\eta_{5}}.\\ {\text{Agg}}_{\eta_{6}} & =\frac{4}{\eta_{6}}+\frac{-3}{\eta_{6}}=\frac{1}{\eta_{6}}.\\ {\text{Agg}}_{\eta_{7}} & =\frac{3}{\eta_{7}}+\frac{-1}{\eta_{7}}=\frac{2}{\eta_{7}}. \end{array}$$
3. Choose the optimal choice based on the maximum aggregate value:
  $$\begin{array}{c} \eta =\underset{i}{\text{max}}\left\{{\text{Agg}}_{\eta_{i}}\right\}=\underset{i}{\text{max}}\{\frac{0}{\eta_{1}},\frac{5}{\eta_{2}},\frac{-1}{\eta_{3}},\frac{-9}{\eta_{4}},\frac{-2}{\eta_{5}},\frac{1}{\eta_{6}},\frac{2}{\eta_{7}}\}=\frac{5}{\eta_{2}}. \end{array}$$
  Therefore, *η*_2_ emerges as the most fitting choice for the project manager role, exhibiting the highest overall value among all candidates. The order of preference is *η*_2_ ≻ *η*_7_ ≻ *η*_6_ ≻ *η*_1_ ≻ *η*_3_ ≻ *η*_5_ ≻ *η*_4_.

#### 5.2 Approach 2: Utilizing Algorithm 2 for project manager selection

Continuing with our example focused on selecting a project manager, we proceed with Approach 2, leveraging Algorithm 2 to further enhance the MCGDM approach within the context of *N*-BHST. The following steps elucidate the decision-making process for identifying the best candidate.

1. Compute the cardinal 3-HS set:
  $$\begin{array}{cc} {\left(F_{\xi }^{1},3\right)}^{•} & =\{\frac{9}{\epsilon_{1}},\frac{7}{\epsilon_{2}},\frac{4}{\epsilon_{3}}\}.\\ {\left(G_{\neg \xi }^{1},3\right)}^{•} & =\{\frac{3}{\epsilon_{1}},\frac{4}{\epsilon_{2}},\frac{4}{\epsilon_{3}}\}.\\ {\left(F_{\xi }^{2},3\right)}^{•} & =\{\frac{5}{\epsilon_{1}},\frac{2}{\epsilon_{2}},\frac{2}{\epsilon_{3}}\}.\\ {\left(G_{\neg \xi }^{2},3\right)}^{•} & =\{\frac{6}{\epsilon_{1}},\frac{7}{\epsilon_{2}},\frac{8}{\epsilon_{3}}\}. \end{array}$$
2. Compute the aggregate 3-HS set:
  $$\begin{array}{c} M_{{\left(F_{\xi }^{1},3\right)}^{*}}=M_{\left(F_{\xi }^{1},3\right)}\times M_{{\left(F_{\xi }^{1},3\right)}^{•}}^{T}=[\begin{array}{cccc} & 2 & 0 & 2\\ & 2 & 1 & 1\\ & 1 & 0 & 0\\ & 1 & 0 & 0\\ & 1 & 2 & 0\\ & 1 & 2 & 1\\ & 1 & 2 & 0 \end{array}][\begin{array}{c} 9\\ 7\\ 4 \end{array}]=[\begin{array}{c} 26\\ 29\\ 9\\ 9\\ 23\\ 27\\ 23 \end{array}]. \end{array}$$
  $$\begin{array}{c} M_{{\left(G_{\neg \xi }^{1},3\right)}^{*}}=M_{\left(G_{\neg \xi }^{1},3\right)}\times M_{{\left(G_{\neg \xi }^{1},3\right)}^{•}}^{T}=[\begin{array}{cccc} & 1 & 1 & 0\\ & 0 & 1 & 0\\ & 0 & 0 & 0\\ & 1 & 2 & 2\\ & 1 & 0 & 2\\ & 0 & 0 & 0\\ & 0 & 0 & 0 \end{array}][\begin{array}{c} 3\\ 4\\ 4 \end{array}]=[\begin{array}{c} 7\\ 4\\ 0\\ 19\\ 11\\ 0\\ 0 \end{array}]. \end{array}$$
  This means:
  $$\begin{array}{cc} {\left(F_{\xi }^{1},3\right)}^{*} & =\{\frac{26}{\eta_{1}},\frac{29}{\eta_{2}},\frac{9}{\eta_{3}},\frac{9}{\eta_{4}},\frac{23}{\eta_{5}},\frac{27}{\eta_{6}},\frac{23}{\eta_{7}}\}. \end{array}$$
  $$\begin{array}{cc} {\left(G_{\neg \xi }^{1},3\right)}^{*} & =\{\frac{7}{\eta_{1}},\frac{4}{\eta_{2}},\frac{0}{\eta_{3}},\frac{19}{\eta_{4}},\frac{11}{\eta_{5}},\frac{0}{\eta_{6}},\frac{0}{\eta_{7}}\}. \end{array}$$
  Similarly,
  $$\begin{array}{c} M_{{\left(F_{\xi }^{2},3\right)}^{*}}=M_{\left(F_{\xi }^{2},3\right)}\times M_{{\left(F_{\xi }^{2},3\right)}^{•}}^{T}=[\begin{array}{cccc} & 1 & 0 & 0\\ & 1 & 1 & 1\\ & 0 & 0 & 0\\ & 1 & 0 & 0\\ & 1 & 0 & 0\\ & 0 & 0 & 1\\ & 1 & 1 & 0 \end{array}][\begin{array}{c} 5\\ 2\\ 2 \end{array}]=[\begin{array}{c} 5\\ 9\\ 0\\ 5\\ 5\\ 2\\ 7 \end{array}]. \end{array}$$
  $$\begin{array}{c} M_{{\left(G_{\neg \xi }^{2},3\right)}^{*}}=M_{\left(G_{\neg \xi }^{2},3\right)}\times M_{{\left(G_{\neg \xi }^{1},3\right)}^{•}}^{T}=[\begin{array}{cccc} & 1 & 2 & 0\\ & 0 & 1 & 0\\ & 1 & 0 & 1\\ & 1 & 2 & 2\\ & 1 & 0 & 2\\ & 1 & 2 & 1\\ & 1 & 0 & 2 \end{array}][\begin{array}{c} 6\\ 7\\ 8 \end{array}]=[\begin{array}{c} 20\\ 7\\ 14\\ 36\\ 22\\ 28\\ 22 \end{array}]. \end{array}$$
  This means:
  $$\begin{array}{cc} {\left(F_{\xi }^{2},3\right)}^{*} & =\{\frac{5}{\eta_{1}},\frac{9}{\eta_{2}},\frac{0}{\eta_{3}},\frac{5}{\eta_{4}},\frac{5}{\eta_{5}},\frac{2}{\eta_{6}},\frac{7}{\eta_{7}}\}. \end{array}$$
  $$\begin{array}{cc} {\left(G_{\neg \xi }^{2},3\right)}^{*} & =\{\frac{20}{\eta_{1}},\frac{7}{\eta_{2}},\frac{14}{\eta_{3}},\frac{36}{\eta_{4}},\frac{22}{\eta_{5}},\frac{28}{\eta_{6}},\frac{22}{\eta_{7}}\}. \end{array}$$
3. Compute the aggregate 3-BHS set for each 3-BHS open set:
  $$\begin{array}{c} {\left(F_{\xi }^{1},G_{\neg \xi }^{1},3\right)}^{*}={\left(F_{\xi }^{1},3\right)}^{*}-{\left(G_{\neg \xi }^{1},3\right)}^{*}=\{\frac{19}{\eta_{1}},\frac{25}{\eta_{2}},\frac{9}{\eta_{3}},\frac{-9}{\eta_{4}},\frac{12}{\eta_{5}},\frac{27}{\eta_{6}},\frac{23}{\eta_{7}}\}. \end{array}$$
  $$\begin{array}{c} {\left(F_{\xi }^{2},G_{\neg \xi }^{2},3\right)}^{*}={\left(F_{\xi }^{2},3\right)}^{*}-{\left(G_{\neg \xi }^{2},3\right)}^{*}=\{\frac{-15}{\eta_{1}},\frac{2}{\eta_{2}},\frac{-14}{\eta_{3}},\frac{-31}{\eta_{4}},\frac{-17}{\eta_{5}},\frac{-26}{\eta_{6}},\frac{-15}{\eta_{7}}\}. \end{array}$$
4. Compute the aggregate value for each alternative:
  $$\begin{array}{cc} {\text{Agg}}_{\eta_{1}} & =\frac{19}{\eta_{1}}+\frac{-15}{\eta_{1}}=\frac{4}{\eta_{1}}.\\ {\text{Agg}}_{\eta_{2}} & =\frac{25}{\eta_{2}}+\frac{2}{\eta_{2}}=\frac{27}{\eta_{2}}.\\ {\text{Agg}}_{\eta_{3}} & =\frac{9}{\eta_{3}}+\frac{-14}{\eta_{3}}=\frac{-5}{\eta_{3}}.\\ {\text{Agg}}_{\eta_{4}} & =\frac{-9}{\eta_{4}}+\frac{-31}{\eta_{4}}=\frac{-40}{\eta_{4}}.\\ {\text{Agg}}_{\eta_{5}} & =\frac{12}{\eta_{5}}+\frac{-17}{\eta_{5}}=\frac{-5}{\eta_{5}}.\\ {\text{Agg}}_{\eta_{6}} & =\frac{27}{\eta_{6}}+\frac{-26}{\eta_{6}}=\frac{1}{\eta_{6}}.\\ {\text{Agg}}_{\eta_{7}} & =\frac{23}{\eta_{7}}+\frac{-15}{\eta_{7}}=\frac{8}{\eta_{7}}. \end{array}$$
5. Choose the optimal choice based on the maximum aggregate value:
  $$\begin{array}{c} \eta =\underset{i}{\text{max}}\left\{{\text{Agg}}_{\eta_{i}}\right\}=\underset{i}{\text{max}}\{\frac{4}{\eta_{1}},\frac{27}{\eta_{2}},\frac{-5}{\eta_{3}},\frac{-40}{\eta_{4}},\frac{-5}{\eta_{5}},\frac{1}{\eta_{6}},\frac{8}{\eta_{7}}\}=\frac{27}{\eta_{2}}. \end{array}$$
  Hence, *η*_2_ emerges as the optimal candidate for the project manager position, exhibiting the highest aggregate value compared to all other candidates. The ranking order is *η*_2_ ≻ *η*_7_ ≻ *η*_1_ ≻ *η*_6_ ≻ *η*_3_ = *η*_5_ ≻ *η*_4_.

## 6 Comparative analysis

This section presents a comparative analysis of the proposed *N*-BHST in conjunction with relevant existing models. Key evaluation factors, including SF (single-argument approximate function), MF (multi-argument approximate function), SO (single opinion), MO (multi-opinion), and BS (bipolarity setting), are taken into account. The objective is to emphasize the versatility and effectiveness of the *N*-BHST concerning these crucial features in comparison to other models in the field.

Table 5 provides a comprehensive comparative overview, illustrating the applicability or limitations of each model with respect to the identified evaluation factors. A check-mark (✓) signifies the successful incorporation of the respective feature, while the symbol (×) denotes its absence or limited implementation.

**Table 5:** Comparison of the *N*-BHST with relevant existing models.

Authors	Models	SF	MF	SO	MO	BS
Fatimah et al. [24]	*N*-S set	✓	×	✓	×	×
Riaz et al. [36]	*N*-ST	✓	×	✓	✓	×
Shabir and Fatima [31] and Mustafa [37]	*N*-BS set	✓	×	✓	×	✓
Mustafa [37]	*N*-BST	✓	×	✓	✓	✓
Musa et al. [48]	*N*-HS set	✓	✓	✓	×	×
Musa and Asaad [49]	*N*-HST	✓	✓	✓	✓	×
Musa [51]	*N*-BHS set	✓	✓	✓	×	✓
Proposed	*N*-BHST	✓	✓	✓	✓	✓

The analysis reveals that the *N*-BHST adeptly deals with all the evaluated features, positioning itself as a comprehensive and versatile approach. Conversely, comparative models have limitations in one or more of these aspects, underscoring the distinct strengths and advancements of the *N*-BHST.

This comparative assessment highlights the superiority of the proposed *N*-BHST, demonstrating its capability to encompass SF, MF, SO, MO, and BS. This differentiation distinguishes it from existing models, emphasizing the significance and relevance of our research and offering valuable insights for the further advancement and adoption of the *N*-BHST in practical applications.

## 7 Concluding remarks

In conclusion, this research has explored the realm of *N*-BHST, an innovative extension of the well-established *N*-HST and HST. Departing significantly from its predecessor, the *N*-BHS set, *N*-BHST introduces a multi-opinion approach to decision-making, enhancing robustness and adaptability. Our analysis has scrutinized various operators within the *N*-BHST framework, shedding light on their interrelationships.

Moreover, we conducted a thorough examination of the application of *N*-BHST in enhancing MCGDM, distinguishing it from existing models. The inclusion of a sensitivity analysis further contributes to the reliability of the proposed *N*-BHST method, ensuring its robustness in various applications. This analysis provides practitioners with valuable insights into how variations in attributes impact decision outcomes, thereby facilitating informed decision-making.

As a compelling direction for future research, we propose extending the envisioned *N*-BHST by integrating fuzzy *N*-BHST. The incorporation of fuzzy logic principles into the current *N*-BHST framework augments the model’s capability to manage uncertainty and imprecision inherent in the evaluation process. This expansion allows for the representation of evaluations with degrees of membership, fostering a more comprehensive and nuanced approach.

Furthermore, our future research endeavors will explore novel hybrid models such as hesitant *N*-BHS set, neutrosophic *N*-BHS set, fermatean *N*-BHS set, and others. By deducing algebraic and topological structures for these models, we aim to provide solutions to various real-world problems characterized by uncertainties. These efforts will contribute to the advancement of decision-making methodologies, enabling practitioners to tackle complex challenges more effectively.

## References

[1] Zadeh L.A., Fuzzy sets, *Information and Control* 8 (1965), 338–353. doi: 10.1016/S0019-9958(65)90241-X

[2] Chung K.L. and Zhong K., A course in probability theory. Academic Press, 2001.

[3] Jaynes E.T., Probability theory: the logic of science. Cambridge University Press, 2003.

[4] Atanassov K., Intuitionistic fuzzy sets, *Fuzzy Sets and Systems* 20 (1986), 87–96. doi: 10.1016/S0165-0114(86)80034-3

[5] Atanassov K., Interval valued intuitionistic fuzzy sets, in: Intuitionistic fuzzy sets, Physica, Springer-Verlag Berlin, Heidelberg, (1999), pp. 139–177.

[6] Gau W.L. and Buehrer D.J., Vague sets, *IEEE Transactions on Systems*, *Man*, *and Cybernetics* 23 (1993), 610–614. doi: 10.1109/21.229476

[7] Pawlak Z., Grzymala-Busse J., Slowinski R. and Ziarko W., Rough sets, *Communications of the ACM* 38 (1995), 88–95. doi: 10.1145/219717.219791

[8] Pawlak Z., Rough sets: theoretical aspects of reasoning about data, Springer Science & Business Media, 2012.

[9] Pawlak Z. and Skowron A., Rudiments of rough sets, *Information Sciences* 177 (2007), 3–27. doi: 10.1016/j.ins.2006.06.003

[10] Pawlak Z. and Skowron A., Rough sets: some extensions, *Information Sciences* 177 (2007), 28–40. doi: 10.1016/j.ins.2006.06.006

[11] Pawlak Z. and Skowron A., Rough sets and Boolean reasoning, *Information Sciences* 177 (2007), 41–73. doi: 10.1016/j.ins.2006.06.007

[12] Gorzałczany M.B., A method of inference in approximate reasoning based on interval-valued fuzzy sets, *Fuzzy Sets and Systems* 21 (1987), 1–17. doi: 10.1016/0165-0114(87)90148-5

[13] Molodtsov D., Soft set theory-first results, *Computers & Mathematics with Applications* 37 (1999), 19–31. doi: 10.1016/S0898-1221(99)00056-5

[14] Saeed M., Saeed M.H., Khalid M. and Mekawy I., Development of hamming and hausdorff distance metrics for cubic intuitionistic fuzzy hypersoft set in cement storage quality control: Development and evaluation, *PLoS ONE* 18 (2023), e0291817. doi: 10.1371/journal.pone.0291817 37747890 PMC10519612

[15] Zhao H., Xiao Q., Liu Z. and Wang Y., An approach in medical diagnosis based on Z-numbers soft set, *PLoS ONE* 17 (2022), e0272203. doi: 10.1371/journal.pone.0272203 36006994 PMC9409603

[16] Jana C., Pal M. and Wang J., A robust aggregation operator for multi-criteria decision-making method with bipolar fuzzy soft environment, *Iranian Journal of Fuzzy Systems* 16 (2019), 1–16.

[17] Jana C., Multiple attribute group decision-making method based on extended bipolar fuzzy MABAC approach, *Computational and Applied Mathematics* 40 (2021), 227. doi: 10.1007/s40314-021-01606-3

[18] Jana C., Garg H., Pal M. et al. MABAC framework for logarithmic bipolar fuzzy multiple attribute group decision-making for supplier selection, *Complex & Intelligent Systems* 10 (2024), 273–288. doi: 10.1007/s40747-023-01108-1

[19] Jana C., Simic V., Pal M., Sarkar B. and Pamucar D., Hybrid multi-criteria decision-making method with a bipolar fuzzy approach and its applications to economic condition analysis, *Engineering Applications of Artificial Intelligence* 132 (2024), 107837. doi: 10.1016/j.engappai.2023.107837

[20] Majumder P., Bhowmik P., Das A., Senapati T., Simic V. and Pamucar D., An intuitionistic fuzzy based hybrid decision-making approach to determine the priority value of indicators and its application to solar energy feasibility analysis, *Optik-International Journal for Light and Electron Optics* 295 (2023), 171492. doi: 10.1016/j.ijleo.2023.171492

[21] Senapati T., An Aczel-Alsina aggregation-based outranking method for multiple attribute decision-making using single-valued neutrosophic numbers, *Complex & Intelligent Systems* 10 (2024), 1185–1199. doi: 10.1007/s40747-023-01215-z

[22] Sarkar A., Moslem S., Esztergár-Kiss D., Akram M., Jin L.S. and Senapati T., A hybrid approach based on dual hesitant q-rung orthopair fuzzy frank power partitioned heronian mean aggregation operators for estimating sustainable urban transport solutions, *Engineering Applications of Artificial Intelligence* 124, 2023, 106505. doi: 10.1016/j.engappai.2023.106505

[23] Alcantud J.C.R. and Laruelle A., Dis&approval voting: a characterization, *Social Choice and Welfare* 43 (2014), 1–10. doi: 10.1007/s00355-013-0766-7

[24] Fatimah F., Rosadi D., Hakim R. and Alcantud J.C.R., *N*-soft sets and their decision making algorithms, *Soft Computing* 22 (2018), 3829–3842. doi: 10.1007/s00500-017-2838-6

[25] Alcantud J.C.R., Feng F. and Yager R.R., An *N*-soft set approach to rough sets, *IEEE Transactions on Fuzzy Systems*, 28 (2020), 2996–3007. doi: 10.1109/TFUZZ.2019.2946526

[26] Akram M., Adeel A. and Alcantud J.C.R., Group decision-making methods based on hesitant N-soft sets, *Expert Systems with Applications* 115 (2019), 95–105. doi: 10.1016/j.eswa.2018.07.060

[27] Akram M., Ali G., Alcantud J.C.R. and Fatimah F., Parameter reductions in *N*‐soft sets and their applications in decision‐making, *Expert Systems* 38 (2021), e12601. doi: 10.1111/exsy.12601

[28] Alcantud J.C.R., The semantics of *N*-soft sets, their applications, and a coda about three-way decision, Information Sciences 606 (2022), 837–852. doi: 10.1016/j.ins.2022.05.084

[29] Akram M., Adeel A. and Alcantud J.C.R., Fuzzy *N*-soft sets: A novel model with applications, *Journal of Intelligent & Fuzzy Systems* 35 (2018), 4757–4771. doi: 10.3233/JIFS-18244

[30] Fatimah F. and Alcantud J.C.R., The multi-fuzzy N-soft set and its applications to decision-making, *Neural Computing and Applications* 33 (2021), 11437–11446. doi: 10.1007/s00521-020-05647-3

[31] Shabir M. and Fatima J., *N*-bipolar soft sets and their application in decision making. 10.21203/rs.3.rs-755020/v1. (2021).

[32] Akram M., Ali G. and Alcantud J.C.R. New decision-making hybrid model: intuitionistic fuzzy *N*-soft rough sets, *Soft Computing* 23 (2019), 9853–9868. doi: 10.1007/s00500-019-03903-w

[33] Akram M., Amjad U. and Davvaz B., Decision-making analysis based on bipolar fuzzy *N*-soft information, *Computational and Applied Mathematics* 40 (2021), 182. doi: 10.1007/s40314-021-01570-y

[34] Mahmood T., Ur Rehman U. and Ali Z., A novel complex fuzzy N-soft sets and their decision-making algorithm, *Complex & Intelligent Systems*, 7 (2021), 2255–2280. doi: 10.1007/s40747-021-00373-2

[35] Rehman U.U. and Mahmood T., Complex intuitionistic fuzzy *N*-soft sets and their applications in decision making algorithm, *Technical Journal* 27 (2022), 95–117.

[36] Riaz M., Çagman N., Zareef I. and Aslam M., *N*-soft topology and its applications to multi-criteria group decision making, *Journal of Intelligent & Fuzzy Systems* 36 (2019), 6521–6536. doi: 10.3233/JIFS-182919

[37] Mustafa H.I., Generalized bipolar-soft sets, generalized bipolar-soft topology and their decision making, *Filomat* 35 (2021), 4587–4611. doi: 10.2298/FIL2113587M

[38] Smarandache F., Extension of soft set to hypersoft set and then to plithogenic hypersoft set, *Neutrosophic Sets and Systems* 22 (2018), 168–170.

[39] Musa S.Y. and Asaad B.A., Bipolar hypersoft sets, *Mathematics* 9 (2021), 1826. doi: 10.3390/math9151826

[40] Musa S.Y. and Asaad B.A., A novel approach towards parameter reduction based on bipolar hypersoft set and its application to decision-making, *Neutrosophic Sets and Systems* 55 (2023), 544–556.

[41] Musa S.Y. and Asaad B.A., Topological structures via bipolar hypersoft sets, *Journal of Mathematics* 2022 (2022), Article ID 2896053.

[42] Musa S.Y. and Asaad B.A., Connectedness on bipolar hypersoft topological spaces, *Journal of Intelligent & Fuzzy Systems* 43 (2022), 4095–4105. doi: 10.3233/JIFS-213009

[43] Saeed M., Rahman A.U., Ahsan M. and Smarandache F., Theory of hypersoft sets: axiomatic properties, aggregation operations, relations, functions and matrices, *Neutrosophic Sets and Systems* 51 (2022), 744–765.

[44] Rana S., Saeed M., Qayyum M. and Smarandache F., Generalized plithogenic whole hypersoft set, PFHSS-Matrix, operators and applications as COVID-19 data structures, *Journal of Intelligent & Fuzzy Systems* 44 (2023), 7797–7820. doi: 10.3233/JIFS-202792

[45] Saeed M., Harl M.I., Saeed M.H. and Mekawy I., Theoretical framework for a decision support system for micro-enterprise supermarket investment risk assessment using novel picture fuzzy hypersoft graph, *PLoS ONE* 18 (2023), e0273642. doi: 10.1371/journal.pone.0273642 36881591 PMC9990954

[46] Smarandache F., New types of soft sets “HyperSoft Set, IndetermSoft Set, IndetermHyperSoft Set, and TreeSoft Set”: An improved version, *Neutrosophic Systems with Applications* 8 (2023), 35–41. doi: 10.61356/j.nswa.2023.41

[47] Sarkar A., Senapati T., Jin L.S., Mesiar R., Biswas A. and Yager R.R., Sugeno–Weber triangular norm-based aggregation operators under T-spherical fuzzy hypersoft context, *Information Sciences* 645 (2023), 119305. doi: 10.1016/j.ins.2023.119305

[48] Musa S.Y., Mohammed R.A. and Asaad B.A., *N*-hypersoft sets: An innovative extension of hypersoft sets and their applications, *Symmetry* 15 (2023), 1795. doi: 10.3390/sym15091795

[49] Musa S.Y. and Asaad B.A., *N*-hypersoft topology: A unified approach for multi-criteria group decision-making, *Journal of Intelligent & Fuzzy Systems* (2024), Submitted. 10.1371/journal.pone.0304016 PMC11108228 38771766

[50] Musa S.Y. and Asaad B.A., Hypersoft topological spaces, *Neutrosophic Sets and Systems* 49 (2022), 397–415.

[51] Musa S.Y., *N*-bipolar hypersoft sets: Enhancing decision-making algorithms, *PLoS ONE* 19 (2024), e.0296396. doi: 10.1371/journal.pone.0296396 38227603 PMC10791010

